# Toward economical application of carbon capture and utilization technology with near-zero carbon emission

**DOI:** 10.1038/s41467-022-35239-9

**Published:** 2022-12-05

**Authors:** Kezia Megagita Gerby Langie, Kyungjae Tak, Changsoo Kim, Hee Won Lee, Kwangho Park, Dongjin Kim, Wonsang Jung, Chan Woo Lee, Hyung-Suk Oh, Dong Ki Lee, Jai Hyun Koh, Byoung Koun Min, Da Hye Won, Ung Lee

**Affiliations:** 1 Clean Energy Research Center, Korea Institute of Science and Technology, Seoul, 02792 Republic of Korea; 2 Department of Chemistry, Kookmin University, Seoul, 02707 Republic of Korea; 3 Division of Energy and Environmental Technology, KIST School, Korea University of Science and Technology (UST), Seoul, 02792 Republic of Korea; 4 Department of Materials Science and Engineering, Korea University, Seoul, 02841 Republic of Korea; 5 KIST-SKKU Carbon-Neutral Research Center, Sungkyunkwan University, Suwon, 16419 Republic of Korea; 6 KU-KIST Graduate School of Energy and Environment, Korea University, Seoul, 02841 Republic of Korea

**Keywords:** Chemical engineering, Electrocatalysis, Carbon capture and storage

## Abstract

Carbon capture and utilization technology has been studied for its practical ability to reduce CO_2_ emissions and enable economical chemical production. The main challenge of this technology is that a large amount of thermal energy must be provided to supply high-purity CO_2_ and purify the product. Herein, we propose a new concept called reaction swing absorption, which produces synthesis gas (syngas) with net-zero CO_2_ emission through direct electrochemical CO_2_ reduction in a newly proposed amine solution, triethylamine. Experimental investigations show high CO_2_ absorption rates (>84%) of triethylamine from low CO_2_ concentrated flue gas. In addition, the CO Faradaic efficiency in a triethylamine supplied membrane electrode assembly electrolyzer is approximately 30% (@−200 mA cm^−2^), twice higher than those in conventional alkanolamine solvents. Based on the experimental results and rigorous process modeling, we reveal that reaction swing absorption produces high pressure syngas at a reasonable cost with negligible CO_2_ emissions. This system provides a fundamental solution for the CO_2_ crossover and low system stability of electrochemical CO_2_ reduction.

## Abstract (web summary)

Carbon capture, utilization and storage technology is limited by the need for a separate CO_2_ capture step. Here, the authors propose a strategy and economic analysis for simultaneous dilute CO_2_ capture from flue gas and direct electrochemical reduction to synthesis gas via reaction swing absorption.

## Introduction

Carbon capture and utilization (CCU) has been recognized as one of the most promising technologies for mitigating climate change seen in decades because of its capacity for large-scale CO_2_ reduction. CCU technology is the only group of technologies that can achieve a net-zero emission target by removing direct and balanced emissions^1^. According to the global roadmap, CCU has the potential to reduce carbon emissions by over 7 gigatons by 2030, and the corresponding market size could reach 800 billion USD^2^. However, the industrial application of CO_2_ conversion technology is still challenging due to its low technological maturity, expensive production cost, and high energy consumption^3^. One way of early settlement of CCU technology is the sequential conversion of CO_2_ via synthesis gas (syngas) generation. Sequential production via syngas has the great advantage of producing various chemicals by adjusting the H_2_-to-CO ratio and employing subsequent reaction processes^4^. However, conventional CO_2_-derived syngas production methods are highly endothermic, which hinders commercialization and consequently leads to question about their ability to reduce CO_2_.

Electrochemical CO_2_ reduction (eCO_2_R) has attracted much attention as an alternative route for sustainable production of CO or syngas^5–8^. eCO_2_R provides a pathway for achieving net-zero emission during syngas production by utilizing renewable energy. Nevertheless, eCO_2_R processes are not economically attractive because CO_2_ conversion through eCO_2_R generally requires expensive CO_2_ capture and product conditioning processes for purified raw material and product supply. Recently, novel efforts have been reported to overcome the cost-inefficiency of eCO_2_R technology by developing new systems excluding pre-/post-conditioning processes, such as utilization of low-concentration CO_2_ (e.g., flue gas)^9–11^ and direct conversion of CO_2_ captured in an amine solution^12,13^. In particular, direct eCO_2_R in an amine solution is a promising strategy because it could eliminate energy-intensive thermal amine regeneration and consume negligible energy for pressurization. Theoretically, the net-zero CO_2_ conversion process can be built based on direct conversion of CO_2_ captured in an amine when the necessary electricity is supplied from a renewable source.

Several attempts have been made to perform eCO_2_R in commercial CO_2_-capturing absorbents (e.g., monoethanolamine, diethanolamine, 2-amino-2-methyl-1-propanol, and their mixtures), and their limitations have been discovered^12,13^. These primary and secondary alkanolamine solvents capture CO_2_ as a carbamate, which inherently contains a strong C-N bond that makes CO_2_ conversion difficult^14^. Chen et al. made an early attempt to demonstrate the possibility of eCO_2_R in a 30 wt.% monoethanolamine solution^12^. This process exhibited a moderate CO Faradaic efficiency (FE) of 38.2%, but the authors also found that the active carbon source for the eCO_2_R was free CO_2_ in the solution, not captured CO_2_ as carbamate. Lee et al. conducted eCO_2_R with captured CO_2_ in a monoethanolamine solution and first observed that the steric properties of a cation in the Helmholtz layer caused the limited performance of eCO_2_R^13^. Addition of electrolytes involving alkali metal cations could constitute a method for improving the eCO_2_R performance. However, this method may be economically unfavorable due to the expensive supply of electrolytes. Li et al. shed light on the direct-capture CO_2_ conversion system by suggesting bicarbonate as a valid option for converting captured CO_2_; this process showed a 37% FE for CO at −100 mA cm^−2^ without the addition of supporting electrolyte^15^, and the performance has further increased to 95% FE for CO at −100 mA cm^−2^ under 4 atm pressure condition, in a recent follow-up study^16^. In this system, KOH is considered to be a currently available CO_2_ absorbent. Although KOH is a promising solvent for CO_2_ capture and conversion, the salt formation and corrosion issues caused by the extremely alkaline condition are considered as major challenges for the commercialization^17–20^. Therefore, a specific method for sustainably supplying bicarbonates from flue gas is required. Most importantly, process design and systematic analyses such as a techno-economic analysis (TEA) or life cycle assessment (LCA) have not been applied in spite of the technological potential of achieving net-zero CO_2_ conversion and producing high-pressure syngas.

Herein, we present an economically feasible and environmentally benign methodology for CO_2_ reduction called a reaction swing absorption (RSA). RSA is a new syngas production method that directly utilizes CO_2_ in flue gas without CO_2_ capture or product separation processes. Throughout this work, we demonstrate experimentally the capability of CO_2_ absorption and direct eCO_2_R of RSA. A conceptual design for RSA is also provided, which shows that RSA can be operated without thermal energy consumption; thus, net-zero CO_2_ emission is possible depending on the energy supply. TEA and LCA are also provided to highlight future research guidelines required for sustainable chemical production via RSA.

## Results

### Reaction swing absorption

**Fig. 1:**
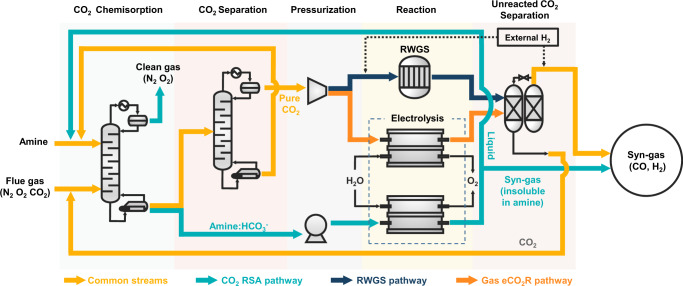
Schematic diagram comparing the RSA pathway with other CCU pathways. Graphical representation of the reaction swing absorption process. The light blue stream represents the RSA pathway. Compared to the RWGS (dark blue) and gas eCO_2_R (orange) pathways, the RSA pathway exhibits a simple process configuration, retains the highest portion of electricity, and is potentially capable of providing a viable CCU solution.

The term RSA refers to processes that selectively separate a target chemical from a gas stream using an absorbent and chemically convert it to desired products. The products are released from the absorbent as the reaction takes place due to solubility differences between reactants and products. For example, the CO_2_ RSA separates CO_2_ from flue gas, and subsequent eCO_2_R in an amine solvent releases high-purity syngas because the product has low solubility in the amine solvent. Figure 1 shows the generalized concept for RSA production of high-pressure syngas (i.e., a CO and H_2_ gas mixture) from CO_2_. RSA consists of chemisorption, pressurization, and electrochemical conversion (e-chemical)^21^ processes. The chemisorption process generates bicarbonate as an absorbent and captures CO_2_ from the flue gas. The bicarbonate solution is pressurized with a pump and then selectively reduced to CO in a zero-gap membrane electrode assembly (MEA) electrolyzer. The unreacted CO_2_ remains in the solution, so a gas product separation process is no longer required.

Figure 1 also describes two conventional syngas production pathways; the reverse water gas shift reaction (RWGS) and gas phase eCO_2_R^9,13,22^. Both pathways exhibit far more complicated process configurations than CO_2_ RSA because they require CO_2_ separation, pressurization, and product separation. In conventional processes, thermal solvent regeneration in CO_2_ separation and high-temperature syngas generation (i.e., RWGS) consume considerable amounts of thermal energy, so CO_2_ reduction in these systems shows limited capability^23^. Additionally, considering the low one-path conversion to CO in eCO_2_R (<50%), an additional process for separating unreacted CO_2_ and recycling it back to the electrolyzer should be considered to maximize carbon utilization^24^. Furthermore, even the most advanced eCO_2_R electrolyzers (e.g., a zero-gap MEA electrolyzer) have experienced systematic problems such as carbonate crossover, which causes system instability and low energy efficiency. The RSA successfully overcomes the abovementioned drawbacks while producing high-pressure and purified syngas. We first describe experimental observations for unit operability of the RSA process and present comprehensive comparisons among CO_2_ RSA, RWGS, and gas phase eCO_2_R via TEA analysis and LCA.

### Amine selection for selective CO_2_ absorption as bicarbonate

**Fig. 2:**
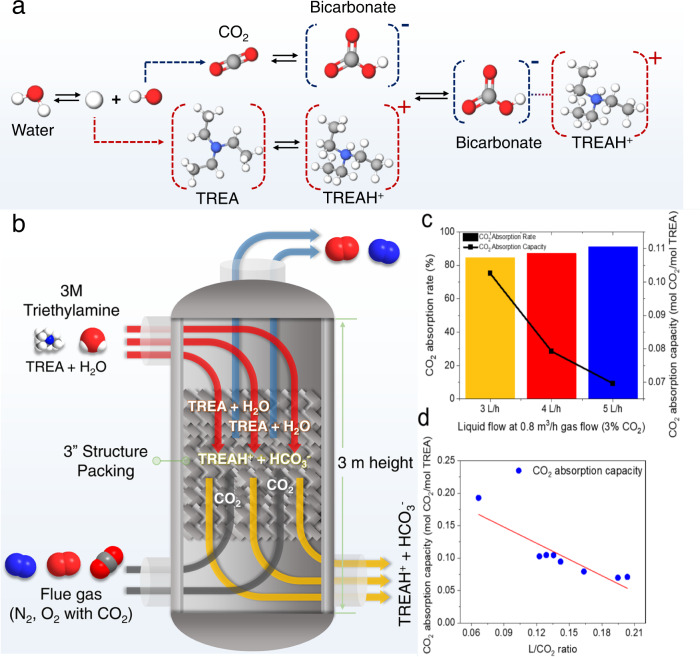
The reaction mechanism for CO_2_ captured in TREA solution and CO_2_ absorption performance in 3 M TREA solvent. **a** A representation of the proposed CO_2_ capture process is shown, where the TREA solution captures CO_2_ by stabilizing the bicarbonate on an equimolar basis. **b** A schematic figure of the CO_2_ absorption column used for the capture experiments. The column is 3 meters in height with a diameter of 3 inches. **c** CO_2_ absorption experiment results with varying TREA flow rates at fixed gas flow rate (0.8 m^3^ h^−1^) and CO_2_ concentration in gas flow (3% CO_2_). **d** Effect of L/CO_2_ ratios on CO_2_ absorption capacity at various values of TREA flow rates (2–5 L h^−1^), gas flow rates (0.5–0.8 m^3^ h^−1^), and CO_2_ concentrations (3–5%). CO_2_ absorption capacity shows a linear correlation with L/CO_2_ ratio, regardless of TREA flow rate, gas flow rate, and CO_2_ concentration.

Alkanolamines are commonly used in industry due to their fast CO_2_ absorption rates and high capacities, but most of them capture CO_2_ as carbamate^25,26^. According to previous studies conducted on eCO_2_R with CO_2_ captured in amine solutions, CO_2_ is not easily released from carbamates due to the strong C-N bonding^13,14^. Thus, it is important to find a new amine that captures CO_2_ in a mild form such as bicarbonate, and directly converts amine-captured CO_2_. After screening various amines, including primary, secondary, and tertiary amines, we found that triethylamine (TREA) is an ideal solvent for both CO_2_ capture and bicarbonate utilization, which contains aliphatic groups but not hydroxyl groups. We confirmed the presence of bicarbonate as the major form of CO_2_ captured in a TREA aqueous solution by nuclear magnetic resonance (NMR) analysis (Fig. S1). In the ^13^C-NMR spectrum of CO_2_ captured in a 3 M TREA/H_2_O solution, a single peak derived from bicarbonate (and/or carbonate) ion was observed. Carbamate species were not observed. Considering that the CO_2_ saturated 3 M TREA/H_2_O is a neutral solution (pH 7–8), it can be deduced that bicarbonate was selectively generated during the absorption of CO_2_ by TREA according to the chemical equilibrium determined by pH^27^. Figure 2a suggests a simplified mechanism for CO_2_ capture in a 3 M TREA solution. The mechanism shows an equimolar reaction between CO_2_ and TREA as bicarbonate generation yields TREA protonation. Note that the theoretical absorption capacity of the TREA is twice that of monoethanolamine, although monoethanolamine has higher reactivity.

The CO_2_ absorption capacity of TREA solvent was measured with a bench scale absorption column equipped with structure packing (Figs. 2b and S2). We measured the CO_2_ absorption rates and capacities at liquid feed/gas CO_2_ feed (L/CO_2_) ratios ranging from 0.07 to 0.21 with synthesized flue gas containing 3–5% CO_2_. Figure 2c shows that the higher the L/CO_2_ ratio at fixed gas flow and CO_2_ concentration, the greater the CO_2_ absorption rate but the less CO_2_ absorption capacity. Figure 2d presents a linear relationship between L/CO_2_ ratio and CO_2_ absorption capacity, regardless of liquid flow (2–5 L h^−1^), gas flow (0.5–0.8 m^3^ h^−1^), and CO_2_ concentration (3–5%). All CO_2_ absorption experiments with TREA in this study exhibit high performance, in terms of CO_2_ absorption rate from 84.5% at fast flue gas flow rate (0.8 m^3^ h^−1^ with 3% CO_2_) to 95.1% at slow flow (0.5 m^3^ h^−1^ with 3% CO_2_) shown in Fig. S2.

### Desirable system configuration for direct conversion of amine-captured CO_2_

**Fig. 3:**
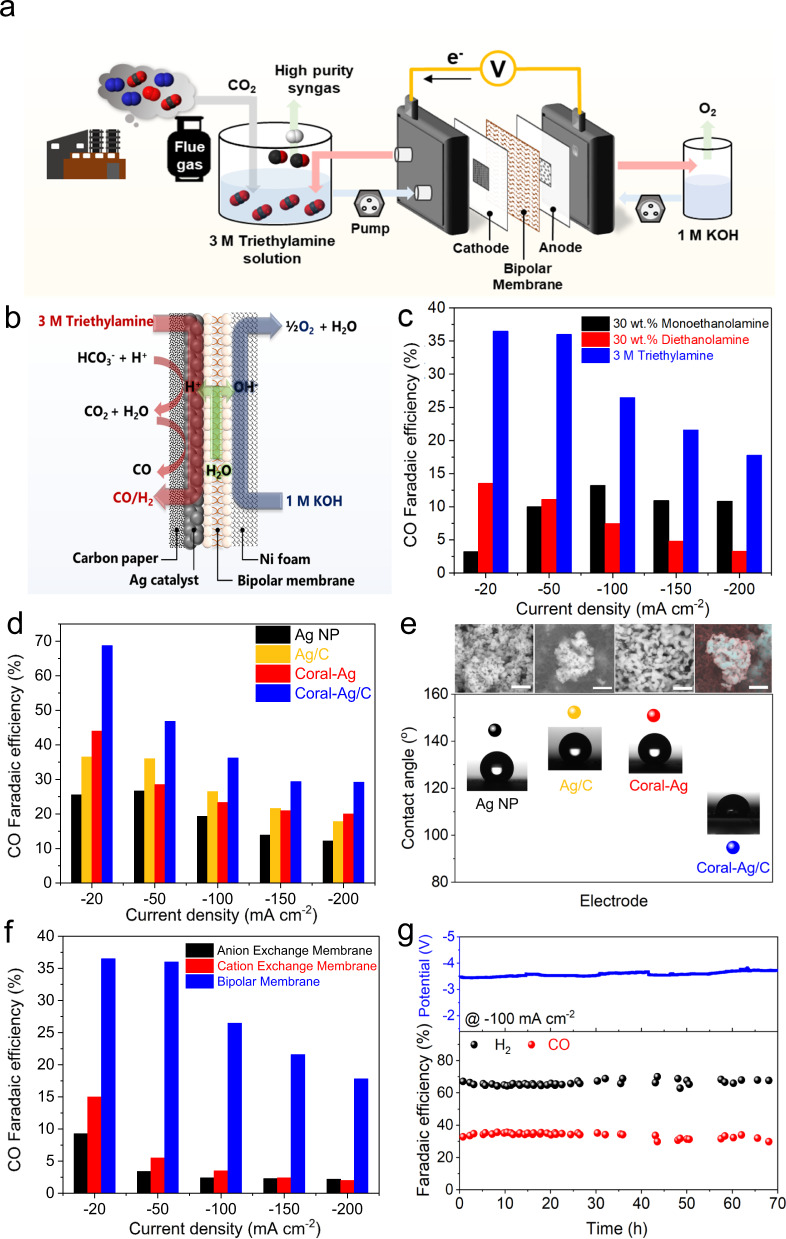
Syngas production with the eCO_2_R system. **a** Schematic of the eCO_2_R system configuration. **b** Schematic of chemical reactions with a cathode, anode, and bipolar membrane. **c** CO FEs for Ag/C measured with various applied current densities in monoethanolamine, diethanolamine, and TREA. **d** CO FEs for Ag NP, Ag/C, coral-Ag, and coral-Ag/C in 3 M TREA. **e** SEM images (scale: 500 nm) and contact angles for all Ag electrodes. **f** CO FEs for Ag/C measured with various membranes, including an anion exchange membrane, cation exchange membrane and bipolar membrane, in 3 M TREA. **g** Long-term CO production represented as the H_2_:CO ratio, CO FEs, and cell voltages (blue) for coral-Ag/C at −100 mA cm^−2^ in 3 M TREA.

CO_2_-captured TREA solution was then supplied to the electrochemical system for CO_2_-derived syngas generation. The eCO_2_R system was designed based on the MEA electrolyzer, which is the most suitable system applied commercially (Fig. 3a)^28,29^. The eCO_2_R system configuration is similar to that of the catholyte-free MEA electrolyzer except that an amine-captured aqueous CO_2_ solution is provided to the cathode instead of humidified CO_2_ gas. Since the targeted product is syngas, Ag nanoparticles (a representative catalyst for CO production)^9,30–34^ on a carbon supporter (Ag/C) were initially selected as a standard catalyst in developing the system. Ni foam was used as the anode while 1 M KOH was supplied as an anolyte. Between the cathode and anode, a bipolar membrane was positioned to separate the two electrodes and supply proton and hydroxide ions to each electrode (Fig. 3b). Gas products from CO_2_ conversion were delivered back to a liquid reservoir with the CO_2_ capture solutions, and then analyzed by gas chromatography (GC). Only gaseous products were detected in this system.

To demonstrate the excellent capability of TREA, eCO_2_R with various CO_2_ absorbents such as monoethanolamine and diethanolamine were compared to that with TREA in the abovementioned system configuration (Figs. 3c and S3a). CO_2_ conversion was evaluated with CO FE at applied constant current densities ranging from −20 to −200 mA cm^−2^ by using chronopotentiometry. CO FEs at all conditions were below 15% when 30 wt.% monoethanolamine and 30 wt.% diethanolamine were used. However, the 3 M TREA outperformed the other amines, demonstrating that it is the best option for a feasible system. Considering that monoethanolamine and diethanolamine transform CO_2_ into carbamates, bicarbonate formation with TREA is the most favorable for direct conversion of CO_2_. Moreover, experiments in which the bicarbonate concentration in TREA was decreased over 6 h of eCO_2_R confirmed that bicarbonate served as the carbon source for CO production (Supplementary Note 3 and Table S1). Initially, there 2.78 M bicarbonate was present in the TREA solution prior to the reaction, and the concentration linearly decreased to 2.47 M as the reaction progressed in 6 h. This result indicated that bicarbonate was utilized as the major CO_2_ source. During the eCO_2_R, CO_2_ was not detected by GC, implying that unreacted CO_2_ was efficiently captured as bicarbonate in the TREA solution (Fig. S4).

Syngas production by the RSA system was further improved by cathode development (Figs. 3d and S3b). In a typical catholyte-free MEA system, CO_2_ is supplied in the gas phase, and thus, hydrophobicity is required for the catalyst electrode to form a three-phase interface, which acts as an active site^35,36^. In contrast, this direct amine-captured CO_2_ conversion system utilizes CO_2_ from the liquid electrolyte, so a hydrophilic catalytic electrode would be preferred for sufficient interaction of the catalyst layer with CO_2_. To simultaneously achieve a suitable electronic structure enabling high CO selectivity for the Ag catalyst as well as hydrophilicity for the carbon supporter, we electrochemically modified the Ag/C into a coral like-structure (coral-Ag/C) through electrochemical oxidation and reduction processes (Fig. 3e)^32^. To identify the effects of the coral-Ag structure and carbon hydrophilicity, additional catalysts, such as (i) Ag nanoparticles (Ag NP) and (ii) coral-structured Ag nanoparticles (coral-Ag), were prepared as a control group, and their characteristics were correlated with their CO production performance (Figs. S5–S10). As expected, the coral-Ag exhibited a higher CO FE than the Ag NPs, and this result clarified that the coral structure was favorable for CO production in the amine-captured CO_2_ conversion system. Randomly connected 3D coral structure with partially oxidized Ag (Ag^+^) near the surface provided suitable surface binding affinities for reaction intermediates^32^. More importantly, the coral-Ag/C catalyst achieved an outstanding CO FE of 70% at a current density of −20 mA cm^−2^ and 30% at a high current density of −200 mA cm^−2^ (Fig. 3d). Considering the moderate performance improvement from Ag NP to Ag coral and from Ag NP to Ag/C, the improved performance of coral-Ag/C implies that there may be other factors to contribute to the performance in addition to the structural modification of Ag and the introduction of carbon support. Thus, we suggest that the hydrophilicity of the electrode is a novel critical factor determining catalytic performance in the RSA system. As shown from contact angle measurements, coral-Ag/C showed the lowest contact angle among the electrodes (Fig. 3e). Similar contact angles for coral-Ag and Ag NPs implied that the electrochemical oxidation and reduction processes were not influenced by the hydrophilicity of Ag but by that of carbon support, as we expected. This influence might derive from increases in the number of oxygen functional groups present on the carbon supporter during electrochemical processes, as observed via X-ray photoelectron spectroscopy (XPS) analysis (Fig. S11). We further compared the eCO_2_R performance according to hydrophobicity of electrodes (Fig. S12). As expected, hydrophobic electrodes showed worse performance with the same Ag catalysts.

System sustainability was secured through selection of an appropriate membrane and confirmed by long-term stability. In the RSA system, since 3 M TREA and 1 M KOH solutions are respectively utilized as catholyte and anolyte, both electrolyte separation for recycling and proton supply for releasing CO_2_ from bicarbonate are important. In this regard, a bipolar membrane is the best option for supplying protons to the cathode and hydroxides to the anode, respectively, while guaranteeing negligible ion transfer between the catholyte and anolyte. In cases with other membranes, such as anion exchange and cation exchange membranes, ion transfer inevitably occurs due to the intrinsic ion-conducting properties, and this ion transfer interferes with the long-term use of the electrolytes. In practice, only bipolar membrane systems have exhibited acceptable CO production performance, while other types of membranes have shown poor CO production performance (Figs. 3f and S3c). It was also confirmed that the bipolar membrane prevented bicarbonate crossover to the anode, since no CO_2_ gas evolution was detected from the anode (Fig. S4). This optimized system configuration (coral-Ag/C cathode and bipolar membrane) demonstrated stable performance with 35% of CO FE, during a 70 h operation of chronopotentiometry experiment at −100 mA cm^−2^ (Fig. 3g).

### Techno-economic analysis and life cycle assessment of RSA

We carried out a comprehensive TEA and an LCA of RSA using a process model based on the abovementioned experimental results. The result was compared with those of conventional processes using the RWGS and gas phase eCO_2_R (Fig. 1) to confirm the superiority of the proposed RSA process. A global sensitivity analysis (GSA) was also performed to assign cost contributions and global warming potentials (GWPs) and eventually to highlight the crucial factors for further improvement of RSA^37^. Current and optimistic scenarios were compared through analysis of several related factors: (i) the electricity generation cost based on different energy sources, (ii) improvements to the bicarbonate electrolyzer, (iii) purchase cost of H_2_ produced from an on-site water electrolysis system as an alternative option to satisfy the H_2_-to-CO ratio of 2 for syngas, and (iv) CO_2_ capture rate of the absorber. H_2_ in syngas comes from purchase, from the CO_2_ electrolyzer as a by-product, or from both. There is competition of H_2_ supply between the bicarbonate electrolyzer and on-site purchase. Therefore, the third factor (purchase cost of H_2_) is added for the sensitivity analysis. Whereas the operating conditions of RWGS and gas eCO_2_R processes are well known, those of the RSA should be optimized due to its early development stage. As a result, the optimal cell voltage, which is pertinent to the CO FE and current density, was found to minimize the break-even price of syngas. The details of the process modeling methodology and TEA and LCA results are explained in Supplementary Note 4.

**Fig. 4:**
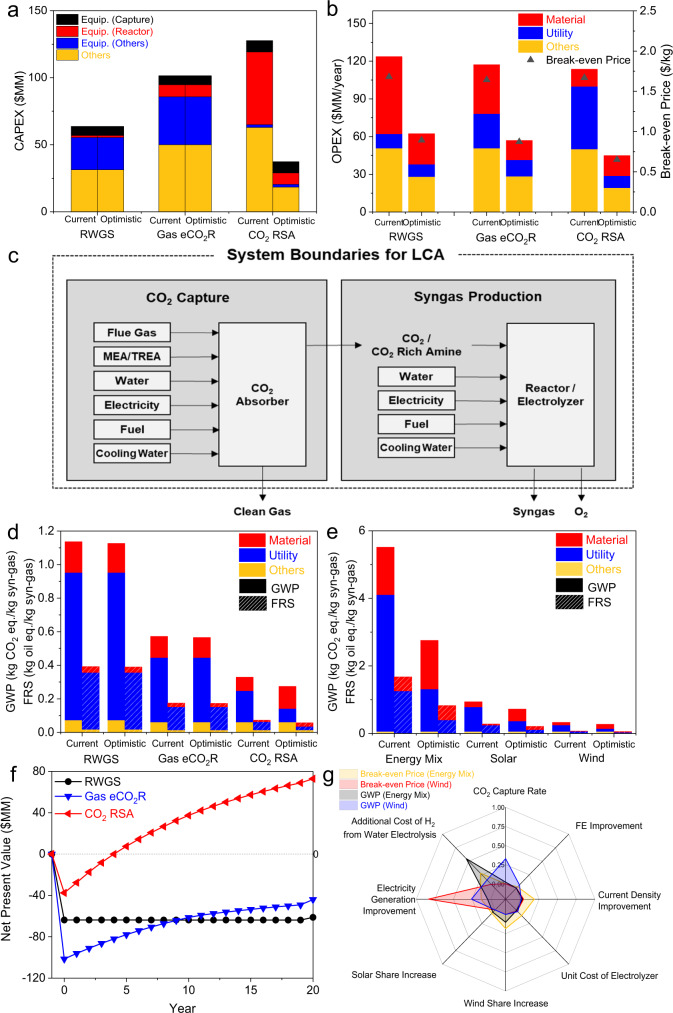
Results of TEA, LCA, and GSA for the wind case. **a** Capital expenditures for three processes. **b** Operating expenditures and break-even prices for syngas from the three processes. **c** Graphical representation of LCA system boundaries. **d** LCA results for the three processes. The left and right bars in each scenario represent global warming potential (GWP) and fossil resource scarcity (FRS), respectively. **e** LCA results for the RSA process with different cases and scenarios. **f** Cash flow charts for an optimistic scenario with a selling price of $0.8/kg syngas. **g** 1st order Sobol indices from global sensitivity analysis.

As illustrated in Figs. 4a, b, S18 and S19, the RSA process outperforms the other two CCU processes, in terms of operating expenditure (OPEX) and the break-even price of syngas in the optimistic scenario, regardless of energy sources. In the current scenario, all three processes have a similar OPEX and break-even price, although the RSA shows an enormous capital expenditure (CAPEX) due to the inefficient bicarbonate electrolyzer. However, current density can be increased at lowered voltage with higher CO FE, as the performance of bicarbonate electrolysis is improved in the optimistic scenario. This improvement considerably reduces CAPEX of the RSA and converts the H_2_ supply from the bicarbonate to water electrolyzer. Considering lower cell voltage in water electrolysis than that in bicarbonate electrolysis, the H_2_ supply change results in the RSA beating other two processes for CAPEX, OPEX, and break-even price in the optimistic scenario. When renewable energy is used, the optimistic break-even prices for syngas will be dropped to $0.65/kg of syngas for wind and $0.56/kg of syngas for solar. These prices can compete with fossil fuel-based syngas processes (Fig. 4f). Although the wind case has a slightly higher syngas price than that in the solar case, wind is a more promising energy source from an environmental perspective (only one-third of the GWP100 value in comparison to the solar case).

Figure 4c presents the system boundaries for LCAs containing CO_2_ sources within the boundaries. Two important impacts for an LCA of CCU technology are GWP100 and fossil resource scarcity (FRS). In the case of GWP100, the RSA process has a lower impact than the other processes in all cases except the current energy mix case (Fig. S20). The GWP100 of the RSA can be minimized to 0.27 kg CO_2_ eq./kg syngas (Fig. 4d) when wind energy is used for electricity generation. However, GWP100 is highly sensitive to the energy source, so this value can be increased up to 5.52 kg CO_2_ eq./kg syngas in the energy mix case (Fig. 4e). The FRS result shows a similar trend to GWP100, because the RSA is highly energy-intensive process.

A GSA was also conducted to support decision-making for establishing appropriate CCU strategies and policies and to provide the priority of research targets. Unlike local sensitivity analyses, such as a one-factor-at-a-time method, GSA changes all uncertain input variables simultaneously and monitors the variances of dependent variables^38^. Thus, a GSA generates Sobol indices, which indicate the impacts of uncertain (input) variables on dependent (output) variables. There are six uncertain variables in the RSA for the solar and wind cases: CO_2_ capture rate, CO FE improvement, current density improvement, unit cost of the electrolyzer, electricity generation improvement, and additional cost (except electricity cost) when H_2_ in syngas is supplied by an on-site water electrolysis system, instead of H_2_ production in the bicarbonate electrolyzer as by-product. For the energy mix case, portions of solar and wind energies in electricity generation mix are added to the uncertain variable set.

Figure 4g compares the 1st order Sobol indices of the break-even price of syngas and the GWP100 for the RSA process under a given range of uncertain variables from current to optimistic scenarios. When wind energy is used for the RSA, the most sensitive economic factor is the electricity generation price (electricity generation improvement in Fig. 4g). Wind energy is well-known for its very low CO_2_ emission, so electricity becomes no longer a major environmental issue. Consequently, uncaptured CO_2_ to be released to air from the absorber is the main contributor to CO_2_ emission when wind energy is used. This result indicates that the near net-zero CO_2_ emission is achievable as the CO_2_ capture rate increases in the chemisorption process. For the energy mix case, no factor dominantly impacts the break-even price. From an environmental perspective, the additional cost except electricity cost to the on-site water electrolysis system is the most significant factor. In other words, the improvement of water electrolysis infrastructure and technology economically attracts this system for H_2_ production and substantially reduces electricity usage. Consequently, the amount of emitted CO_2_ in the energy mix case can be largely decreased by this improvement. The detail TEA, LCA, and GSA results for different scenarios and cases can be seen in Supplementary Note 4.

## Discussion

We report RSA as a potential net-zero CO_2_ emission methodology for syngas production from CO_2_. By utilizing a bicarbonate-generating amine solvent, TREA, RSA successfully captures CO_2_ from flue gas and selectively reduces it to syngas via direct electrochemical CO_2_ reduction. The CO_2_ absorption capability of TREA was measured with a bench scale absorber and a scalable system configuration for eCO_2_R, and this further supported the feasibility of RSA in industrial applications. After optimization of variables in the eCO_2_R system, such as catalyst, electrolyte, and membrane, RSA produced high-purity syngas composed of 30 to 70% CO with a balance of H_2_, depending on applied current densities ranging from −200 mA cm^−2^ to −20 mA cm^−2^. It is worth noting that the impact of trace amounts of impurities, such as NO_3_^-^, on the bicarbonate electrolysis system should be investigated before upscaling the process^16^. A TEA based on the experimental results showed that RSA is the most promising CCU process with the lowest CO_2_ emissions, and it outperforms current CCU processes.

**Fig. 5:**
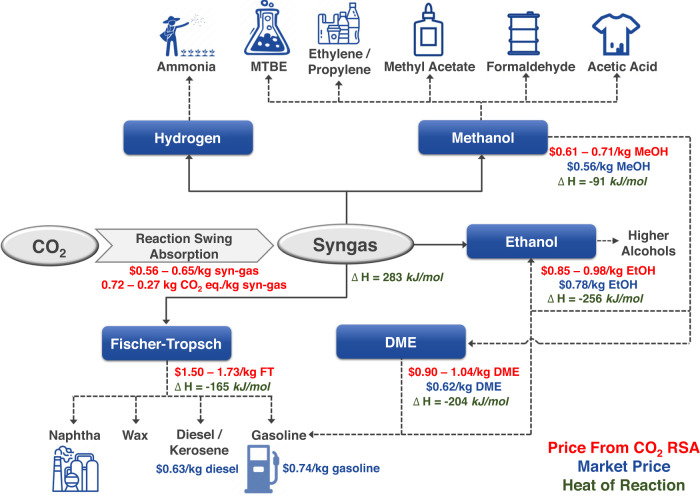
Syngas as a versatile intermediate chemical having a competitive price when using the eco-friendly CO_2_ RSA process. Estimated levelized cost of target chemicals produced from consecutive RSA and downstream processes^39^ (red), current market prices of chemicals (blue), and heat of reactions of syngas production and downstream reactions^40,41^ (green). (Market prices from industries^42–44^ and a report^45^.

CCU technology has been studied substantially but commercialization is still limited by many challenges and difficulties originating from the high stability of CO_2_. If syngas, a product of the endothermic reactions of CO_2_, can be produced economically, then it is possible that CCU technology can be industrialized because the downstream processes are exothermic (Fig. 5). For example, methanol, ethanol, and dimethyl ether synthesis and the Fischer-Tropsch reaction are highly exothermic, implying that these downstream processes can be operated without excessive energy consumption. Considering the technological maturity of downstream processes utilizing syngas, we believe that RSA can change the paradigm for chemical processes by realizing the eco-friendly and cost-effective production of syngas from CO_2_. In Fig. 5, energy-intensive syngas production accounts for the highest proportion in terms of cost and energy consumption. Therefore, employing an environmentally benign and economically feasible process of syngas production could accelerate the commercialization of CCU technology. Such acceleration may be achieved through the RSA process proposed in this study because it requires neither an energy-intensive step for amine solvent regeneration nor a separation system of unreacted CO_2_. According to the modeling results of this study, it is crucial to improve the bicarbonate electrolyzer used in RSA and integrate the system with renewable energy sources in order to make the price highly competitive with that of fossil fuel-based syngas and achieve net-zero CO_2_ emission.

## Methods

### CO_2_ capture rate measurement

The CO_2_ capture rate of 3 M TREA solution is measured in a pilot scale absorber. A micro gear pump (GA-V21) and a mass flow controller are used to control the simulated flue gas and absorbent flow rates. The 3–5% of simulated flue gas are prepared using 99.99% CO_2_, N_2_, and air. The flue gas and absorbent are count currently contacted in an absorber equipped with 3-inch Sulzer structure packing (DX) in order to maximize the mass transfer between the gas and liquid. We calculated CO_2_ capture rate by measuring inlet and outlet CO_2_ flow rates. To achieve the steady state condition of the absorber operation, each experiment lasted at least 45 min. More Detail information is available in Supplementary Note 1.

### Catalyst preparation and characterization

For all Ag electrodes, a carbon paper (AvCsrb MGL190) with 200 nm of Ag primary layer, which was deposited by e-beam evaporator (UL VAL Inc.) with 3 A s^−1^ deposition rate under 10^−6^–10^−7^ Torr of vacuum condition, was used as a substrate. The Ag nanoparticle (Ag NP) catalysts (Alfa Aesar, 99.9%) were basically deposited on the prepared substrate by spraying-coating method and modified by mixing carbon or by conducting electrochemical structural tuning. They were named according to their composition and structure such as Ag NP, Ag/C, coral-Ag, and coral-Ag/C. First, Ag NP electrode was prepared by spraying of ink solution which was composed of Ag NP (120 mg), isopropanol (5 ml), and 5 wt.% Nafion perfluorinated resin solution (128 µl) with catalyst loading amount 1 mg cm^−2^. The only difference for Ag/C electrode was that Ag NP was mixed with Ketjen black 600JD with mass ratio 75% of Ag NP in the catalyst ink solution. Coral-Ag and coral-Ag/C electrodes were respectively prepared by oxidizing of Ag NP and Ag/C in the 0.1 M Ar-saturated KCl (Sigma-Aldrich, >99%) solution at 0.3 V (vs Ag/AgCl (3 M KCl) reference and Pt foil counter electrode for three-electrode system) for 12 h and then reducing them in 0.1 M KHCO_3_ at −1.2 V (vs Ag/AgCl (3 M KCl)) for 30 min as previously reported method^32^. The morphology of the prepared Ag catalysts were characterized by scanning electron microscope (SEM) and energy dispersive X-ray spectroscopy (EDX) mapping (Hitachi Regulus 8230 with UHR cold type field emitter gun). The chemical structure of Ag electrodes was analyzed by XPS spectra using Nexsa (Thermo Fisher Scientiffic) with a monochromated Al-Kα (1486.6 eV) source. To understand the effect of the hydrophilicity/phobicity of the prepared Ag electrodes, contact angle was measured by a contact angle meter (DSA 25, Kruss) with 2.0 µl of DI water.

### Electrochemical measurement

All electrochemical CO_2_R were performed in a commercial MEA electrolyzer (complete 5 cm^2^ CO_2_ electrolyzer, Dioxide materials). The as-prepared Ag electrode and nickel foam (200 mm length × 300 mm width × 1.6 mm thickness, MTI Korea) were used as the cathode and anode, respectively. The bipolar membrane (Fumasep FBM) was positioned between cathode and anode. The active area was controlled as 1 cm^2^. 3 M TREA (Sigma-Aldrich, 99%) was used as the CO_2_ capture solution and 1 M KOH (Sigma-Aldrich, >90%) was employed as anolyte. It is assumed that the TREA solution absorbs only CO_2_ from the flue gas. Therefore, 3 M TREA solution was saturated with CO_2_ for 1 h before eCO_2_R. The catholyte and anolytes were respectively provided from liquid reservoirs to MEA electrolyzer and continuously circulated by using a peristaltic pump. For comparison of CO_2_ captured solution, the 30 wt.% monoethanolamine or 30 wt.% diethanolamine were supplied instead of 3 M TREA. The eCO_2_R of all prepared Ag electrodes were examined by chronopotentiometry using a potentiostat (VSP, Biologic with booster 20 A) with current density range from −20, −50, −100, −150, to −200 mA cm^−2^. The gas products from the eCO_2_R were periodically quantified by online GC with a thermal conductivity detector and a flame ionization detector (Agilent 7890). MolSieve 5 A (6FT, Agilent) and Hayesep D (11FT, Agilent) packed columns were equipped, and ultrapure Ar (99.9999%) was used as the carrier gas. The products from CO_2_ conversion were CO and H_2_, and the total FE was confirmed to be ~100%. The FE of the products (i.e., H_2_ and CO) was calculated by the ratio of each partial current of the products to the total current.

## Supplementary information

Supplementary Information

Peer Review File

## Data Availability

The data supporting the findings of this study are available within the article and its Supplementary Information file.

## Code availability

The codes generated in this study are available upon reasonable request to the corresponding authors.

## References

[1] International Energy Agency. *CCUS in clean energy transitions*. https://www.iea.org/reports/ccus-in-clean-energy-transitions (2020).

[2] Global CO2 Initiative. *Global roadmap for implementing CO*_*2*_*utilization*. https://deepblue.lib.umich.edu/handle/2027.42/150624 (2016).

[3] Hepburn C (2019). The technological and economic prospects for CO_2_ utilization and removal. Nature.

[4] Hernandez S (2017). Syngas production from electrochemical reduction of CO_2_: current status and prospective implementation. Green. Chem..

[5] Wen J (2017). Understanding activity and selectivity of metal-nitrogen-doped carbon catalysts for electrochemical reduction of CO_2_. Nat. Commun..

[6] Lee JH (2019). Tuning the activity and selectivity of electroreduction of CO_2_ to synthesis gas using bimetallic catalysts. Nat. Commun..

[7] Zheng T (2019). Large-scale and highly selective CO_2_ electrocatalytic reduction on nickel single-atom catalyst. Joule.

[8] Hua Y, Zhang B, Hao W, Gao Z (2022). Boosting CO desorption on dual active site electrocatalysts for CO_2_ reduction to produce tunable syngas. Cell Rep. Phys. Sci..

[9] Kim D (2021). Electrocatalytic reduction of low concentrations of CO_2_ gas in a membrane electrode assembly electrolyzer. ACS Energy Lett..

[10] Choi B-U, Tan YC, Song H, Lee KB, Oh J (2021). System design considerations for enhancing electroproduction of formate from simulated flue gas. ACS Sustain. Chem..

[11] Alerte T (2021). Downstream of the CO_2_ electrolyzer: assessing the energy intensity of product separation. ACS Energy Lett..

[12] Chen L (2017). Electrochemical reduction of carbon dioxide in a monoethanolamine capture medium. ChemSusChem.

[13] Lee G (2021). Electrochemical upgrade of CO_2_ from amine capture solution. Nat. Energy.

[14] Sullivan I (2021). Coupling electrochemical CO_2_ conversion with CO_2_ capture. Nat. Catal..

[15] Li T (2019). Electrolytic conversion of bicarbonate into CO in a flow cell. Joule.

[16] Zhang Z (2022). Porous metal electrodes enable efficient electrolysis of carbon capture solutions. Energy Environ. Sci..

[17] Galvez-Vazquez MdJ (2020). Environment matters: CO2RR electrocatalyst performance testing in a gas-fed zero-gap electrolyzer. ACS Catal..

[18] Dinh C-T, Arquer FPGD, Sinton D, Sargent EH (2018). High rate, selective, and stable electroreduction of CO_2_ to CO in basic and neutral media. ACS Energy Lett..

[19] Endrodi B (2019). Multilayer electrolyzer stack converts carbon dioxide to gas products at high pressure with high efficiency. ACS Energy Lett..

[20] Jeanty P (2018). Upscaling and continuous operation of electrochemical CO_2_ to CO conversion in aqueous solutions on silver gas diffusion electrodes. J. CO2 Util..

[21] Na J (2019). General technoeconomic analysis for electrochemical coproduction coupling carbon dioxide reduction with organic oxidation. Nat. Commun..

[22] Joo O-S (1999). Carbon dioxide hydrogenation to form methanol via a reverse-water-gas-shift reaction (the CAMERE process). Ind. Eng. Chem. Res..

[23] Artz J (2018). Sustainable conversion of carbon dioxide: an integrated review of catalysis and life cycle assessment. Chem. Rev..

[24] Rabinowitz JA, Kanan MW (2020). The future of low-temperature carbon dioxide electrolysis depends on solving one basic problem. Nat. Commun..

[25] Heldebrant DJ (2017). Water-lean solvents for post-combustion CO_2_ capture: fundamentals, uncertainties, opportunities, and outlook. Chem. Rev..

[26] Lee U (2016). Techno-economic optimization of a green-field post-combustion CO_2_ capture process using superstructure and rate-based models. Ind. Eng. Chem. Res..

[27] Mani F, Peruzzini M, Stoppioni P (2006). CO_2_ absorption by aqueous NH_3_ solutions: speciation of ammonium carbamate, bicarbonate and carbonate by a ^13^C NMR study. Green. Chem..

[28] Higgins D, Hahn C, Xiang C, Jaramillo TF, Weber AZ (2018). Gas-diffusion electrodes for carbon dioxide reduction: a new paradigm. ACS Energy Lett..

[29] Gabardo CM (2019). Continuous carbon dioxide electroreduction to concentrated multi-carbon products using a membrane electrode assembly. Joule.

[30] Buckley AK (2021). Approaching 100% selectivity at low potential on Ag for electrochemical CO_2_ reduction to CO using a surface additive. ACS Catal..

[31] Rosen BA (2011). Ionic liquid-mediated selective conversion of CO_2_ to CO at low overpotentials. Science.

[32] Lee WH (2020). Highly selective and scalable CO_2_ to CO - electrolysis using coral-nanostructured Ag catalysts in zero-gap configuration. Nano Energy.

[33] Clark EL (2019). Influence of atomic surface structure on the activity of Ag for the electrochemical reduction of CO_2_ to CO. ACS Catal..

[34] Nguyen DL, Kim T, Hwang YJ, Won DH (2020). Progress in development of electrocatalyst for CO_2_ conversion to selective CO production. Carbon Energy.

[35] Xing Z, Hu L, Ripatti DS, Hu X, Feng X (2021). Enhancing carbon dioxide gas-diffusion electrolysis by creating a hydrophobic catalyst microenvironment. Nat. Commun..

[36] Sa YJ (2020). Catalyst–electrolyte interface chemistry for electrochemical CO_2_ reduction. Chem. Soc. Rev..

[37] Sobol IM (2020). Global sensitivity indices for nonlinear mathematical models and their Monte Carlo estimates. Math. Comput. Simul..

[38] Kim J (2021). Learning the properties of a water-lean amine solvent from carbon capture pilot experiments. Appl. Energy.

[39] Schemme S (2020). H_2_-based synthetic fuels: a techno-economic comparison of alcohol, ether and hydrocarbon production. Int. J. Hydrog. Energy.

[40] Koybasi HH, Hatipoglu C, Avci AK (2021). Sustainable DME synthesis from CO_2_-rich syngas in a membrane assisted reactor–microchannel heat exchanger system. J. CO2 Util..

[41] Ali SS, Ali SS, Tabassum N (2022). A review on CO_2_ hydrogenation to ethanol: Reaction mechanism and experimental study. J. Environ. Chem. Eng..

[42] Methanex. *Methanex posts regional contract methanol prices for Europe, North America, Asia and China*. https://www.methanex.com/our-business/pricing (2022).

[43] TradingEconomics. *Commodities*. https://tradingeconomics.com/commodities (2022).

[44] GlobalPetrolPrice.com. *Retail energy price data*. https://www.globalpetrolprices.com (2022).

[45] Yang, A. S., Antrassian, C. V. & Kurtzman, J. D. *Production of Dimethyl Ether (DME) for Transportation Fuel*. https://repository.upenn.edu/cbe_sdr/119/ (2020).

