# Supplementary material for: Toward economical application of carbon capture and utilization technology with near-zero carbon emission
Source: Nat Commun. 2022 Dec 5;13:7482. doi: 10.1038/s41467-022-35239-9 (PMC9722933; doi:10.1038/s41467-022-35239-9)
Supplement: Supplementary file 1 — Supplementary Information [file 41467_2022_35239_MOESM1_ESM.docx]

**Supplementary Information**

**Toward economical application of carbon capture and utilization technology with near zero carbon emission**

Kezia Megagita Gerby Langie^1,2†^, Kyungjae Tak^1,†^, Changsoo Kim^1^, Hee Won Lee^1,3^, Kwangho Park^1^, Dongjin Kim^1,4^, Wonsang Jung^1,3^, Chan Woo Lee^2^, Hyung-Suk Oh^1,5^, Dong Ki Lee^1,6^, Jai Hyun Koh^1,3^, Byoung Koun Min^1,6^, Da Hye Won^1,3,*^, and Ung Lee^1,3,6*^

^1^Clean Energy Research Center, Korea Institute of Science and Technology, Seoul 02792, Republic of Korea

^2^Department of Chemistry, Kookmin University, Seoul 02707, Republic of Korea

^3^Division of Energy and Environmental Technology, KIST School, Korea University of Science and Technology (UST), Seoul 02792, Republic of Korea

^4^Department of Materials Science and Engineering, Korea University, Seoul 02841, Republic of Korea

^5^KIST-SKKU Carbon-Neutral Research Center, Sungkyunkwan University, Suwon 16419, Republic of Korea

^6^KU-KIST Graduate School of Energy and Environment, Korea University, Seoul 02841, Republic of Korea

^*^Corresponding author(s): Da Hye Won (dahye0803@kist.re.kr) and Ung Lee (ulee@kist.re.kr)

^†^These authors contributed equally to this work.

**Table of contents**

[Supplementary Notes 1: TREA CO_2_ capture experiment 3](#_Toc113450795)

[Supplementary Notes 2: Characterizations 3](#_Toc113450796)

[Supplementary Notes 3: Quantification of bicarbonate in TREA aqueous solution by precipitation method 4](#_Toc113450797)

[Table S1. The change of concentration of bicarbonate in TREA solution during CO_2_ reduction. 4](#_Toc113450798)

[Figure S1. The presence of bicarbonate in 3 M TREA 5](#_Toc113450799)

[Figure S2. CO_2_ absorption experiments 5](#_Toc113450800)

[Figure S3. CO FE and H_2_ FE for different catalysts with various membranes 6](#_Toc113450801)

[Figure S4. GC chromatogram of gas samples under various conditions 7](#_Toc113450802)

[Figure S5. Chemical state of the prepared Ag catalysts 7](#_Toc113450803)

[Figure S6. XRD spectra of the prepared Ag catalysts 8](#_Toc113450804)

[Figure S7. Morphology of coral-Ag/C 8](#_Toc113450805)

[Figure S8. Structure of the prepared Ag catalysts 9](#_Toc113450806)

[Figure S9. Applied potential for eCO_2_R 10](#_Toc113450807)

[Figure S10. The eCO_2_R performance of substrate (Ag e-beam) 10](#_Toc113450808)

[Figure S11. Differences in carbon supporter by electrochemical process for coral-structure 11](#_Toc113450809)

[Figure S12. Hydrophilic/phobic effect on CO production performance 11](#_Toc113450810)

[Supplementary Note 4: Comparison of CCU processes through modeling approach 12](#_Toc113450811)

[4.1. Description of three CCU processes 12](#_Toc113450812)

[Figure S13. Flowsheets of CO_2_ conversion processes 15](#_Toc113450813)

[Figure S14. Experiment data and future expectation of the bicarbonate electrolysis performance 16](#_Toc113450814)

[4.2. Model development for techno-economic analysis, life cycle assessment, and global sensitivity analysis 17](#_Toc113450815)

[Table S2. Design basis for process simulations. 17](#_Toc113450816)

[Table S3. Parameters for techno-economic evaluation. 20](#_Toc113450817)

[Figure S15. Components and their proportion on total production cost. 20](#_Toc113450818)

[Figure S16. Scheme of global sensitivity analysis. 22](#_Toc113450819)

[4.3. Modeling results 23](#_Toc113450820)

[Table S4. Parameters values used for global sensitivity analyses. 23](#_Toc113450821)

[Figure S17. Current and optimistic electricity generation costs from different energy sources 24](#_Toc113450822)

[Figure S18. Capital investment of three processes under various energy sources 27](#_Toc113450823)

[Figure S19. Operating cost and break-even price of three processes under various energy sources 28](#_Toc113450824)

[Figure S20. LCA result of three processes under various energy sources. (a) RWGS process. (b) Gas eCO_2_R process. (c) CO_2_ RSA process. 31](#_Toc113450825)

[Figure S21. RIC and GWP for syngas-based chemical production from CCU pathways in the literature 32](#_Toc113450826)

[Figure S22. GSA result of the RSA process in the energy mix case 34](#_Toc113450827)

[Figure S23. GSA result of the RSA process in the solar case 35](#_Toc113450828)

[Figure S24. GSA result of the RSA process in the wind case 36](#_Toc113450829)

[4.4. Reference 37](#_Toc113450830)

## **Supplementary Notes 1: Triethylamine CO_2_ capture experiment**

To confirm the CO_2_ captured form in triethylamine (TREA) solution, ^13^C and ^1^H nuclear magnetic resonance spectroscopy (NMR, 400 MHz NMR, Bruker advanced II HD) are analyzed. We can confirm the bicarbonate is the major form of CO_2_ captured in TREA.

The CO_2_ capture experiment is conducted by a pilot scale absorption column. The absorber is 3 meter high with 5 L bottom tank and condenser. The absorber is equipped with 3” Sulzer structure packing in order to maximize vapor-liquid mass transfer. Flue gas is introduced to the bottom of the absorber while 3 M TREA is fed to top. The flue gas after CO_2_ removal by amine solvent is vented to the top of the condenser, and outlet CO_2_ concentration is detected by CO_2_ probe (Optima7). 15 thermo-couples are installed in the absorber and the temperature profile is also monitored. The six experiment conditions are evaluated, reflecting different flue gas flow rate (0.5–0.8 m^3^ h^-1^), CO_2_ concentration (3–5%), and flow rate of amine solvent (2–5 L h^-1^).

**Supplementary Notes 2: Characterizations**

The morphology of the prepared Ag electrodes is observed by scanning electron microscopy (SEM) and transmission electron microscopy (TEM) analysis. SEM images are collected with backscarttered electron (BSE) image, which can observe the topography of samples by Hitachi Regulus 8230 with UHR cold type field emitter gun. TEM analysis is further conducted the Ag coral structure by using a TitanTM 80-300 at an accelerating voltage of 200 kV. To understand the chemical structure of Ag in prepared Ag electrodes, Ag 3d and C 1s are analyzed by X-ray photoelectron spectroscopy (XPS, Nexsa Thermo Fisher Scientific) with a monochromated Al-Kα (1486.6 eV) source. The crystalline structures of the prepared Ag electrodes are analyzed by X-ray diffraction (XRD, LynxEye D8 Advance) with energy dispersive detector.

To observe the change of hydrophobicity/philicity during electrochemical process into coral structure, we analyze the contact angle of prepared the Ag electrodes. Water contact angle can be measured by a contact angle meter (DSA 25, Kruss) with 2.01 µl of D.I. water.

## **Supplementary Notes 3: Quantification of bicarbonate in TREA aqueous solution by precipitation method**

For quantification of bicarbonate in TREA solution, we applied the cation substitution chemical reaction, causing a precipitation of bicarbonate into insoluble carbonate. When soluble CaCl_2_ is added to the bicarbonate solution, solid CaCO_­3_ is precipitated as below description. We can track the bicarbonate concentration by measuring the amount of CaCO_3_.

$\mathrm{CO}_{2}\left( g \right)+H_{2}O\left( l \right)+TR\mathrm{EA}\left( l \right)\to{TR\mathrm{EAH}}^{+}-\mathrm{HCO}_{3}^{-}(aq)$ (1)

${TR\mathrm{EAH}}^{+}-\mathrm{HCO}_{3}^{-}\left( \mathrm{aq} \right)+0.5\mathrm{CaCl}_{2}\left( s \right)\to TR\mathrm{EA}H^{+}-\mathrm{Cl}^{-}\left( \mathrm{aq} \right)+\frac{1}{2}\mathrm{Ca}\left( \mathrm{HC}O_{3} \right)_{2}(aq)$ (2)

$\frac{1}{2}\mathrm{Ca}\left( \mathrm{HC}O_{3} \right)_{2}\left( \mathrm{aq} \right)\to\frac{1}{2}\mathrm{CaCO}_{3}\left( s, \mathrm{precipitation} \right)+\frac{1}{2}H_{2}O\left( l \right)+\frac{1}{2}CO_{2}(g)$ (3)

To experimentally confirm the origin of carbon source for syngas production, the bicarbonate concentration in TREA solution according to electrochemical CO_2_ reduction reaction is tracked over time. We collect 5 samples of TREA solutions during 6 h reaction, consists of pre-reaction, during CO_2_ reduction reaction samples, and after 6 h of CO_2_ reduction reaction (Table S1). Thus, based on the Table S1, we calculate the consumed CO_2_ is 0.013 mol in bicarbonate. This value is almost similar with the CO_2_ used to produce CO (0.015 mol for 6 h) which is analyzed by gas chromatography.

## **Table S1.** The change of concentration of bicarbonate in TREA solution during CO_2_ reduction.

| Sample | V_sample_ | V_electrolyte_^a^ | CaCO_3_ | HCO_3_^-^ | HCO_3_^-^ |
| --- | --- | --- | --- | --- | --- |
|  | (ml) | (ml) | (g) | (g) | (M) |
| Pre reaction | 1 | 50 | 0.1390 | 0.169 | 2.78 |
| 1.5 h | 1 | 50 | 0.1374 | 0.168 | 2.75 |
| 3 h | 1 | 46 | 0.1324 | 0.161 | 2.65 |
| 4.5 h | 1 | 42 | 0.1299 | 0.158 | 2.6 |
| 6 h | 1 | 38 | 0.1236 | 0.151 | 2.47 |

^a^ 4 ml of electrolyte was collected each time for analysis.

**
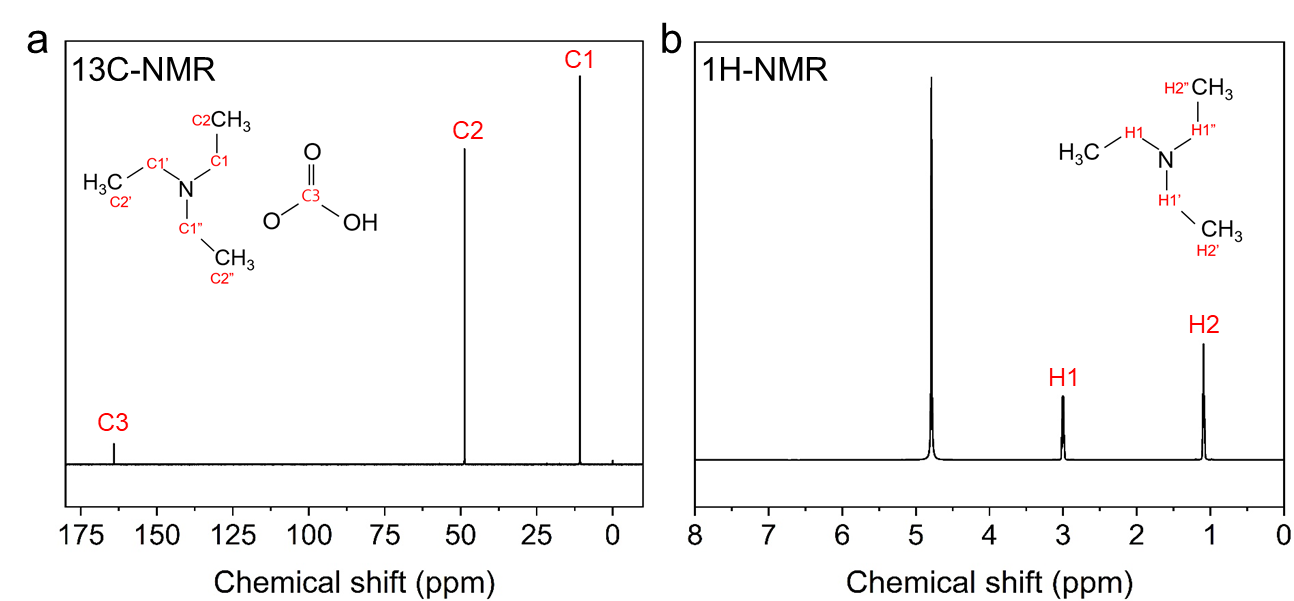
**

^1^H-NMR

^13^C-NMR

## **Figure S1. The presence of bicarbonate in 3 M TREA**. (a) ^13^C-NMR and (b) ^1^H-NMR analysis of CO_2_ captured 3 M TREA solution.


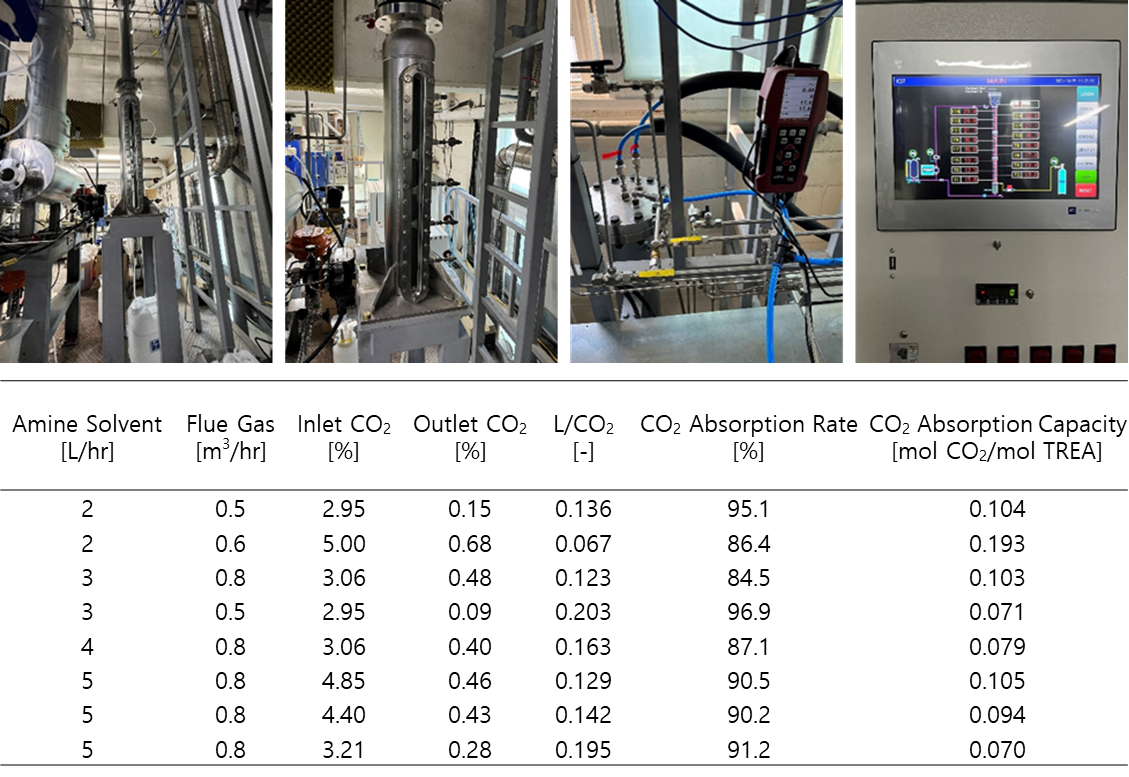


## **Figure S2. CO_2_ absorption experiments.** The photo images of absorption column and system. (Inset table) Experimental result of CO_2_ absorption in 3 M TREA solution.


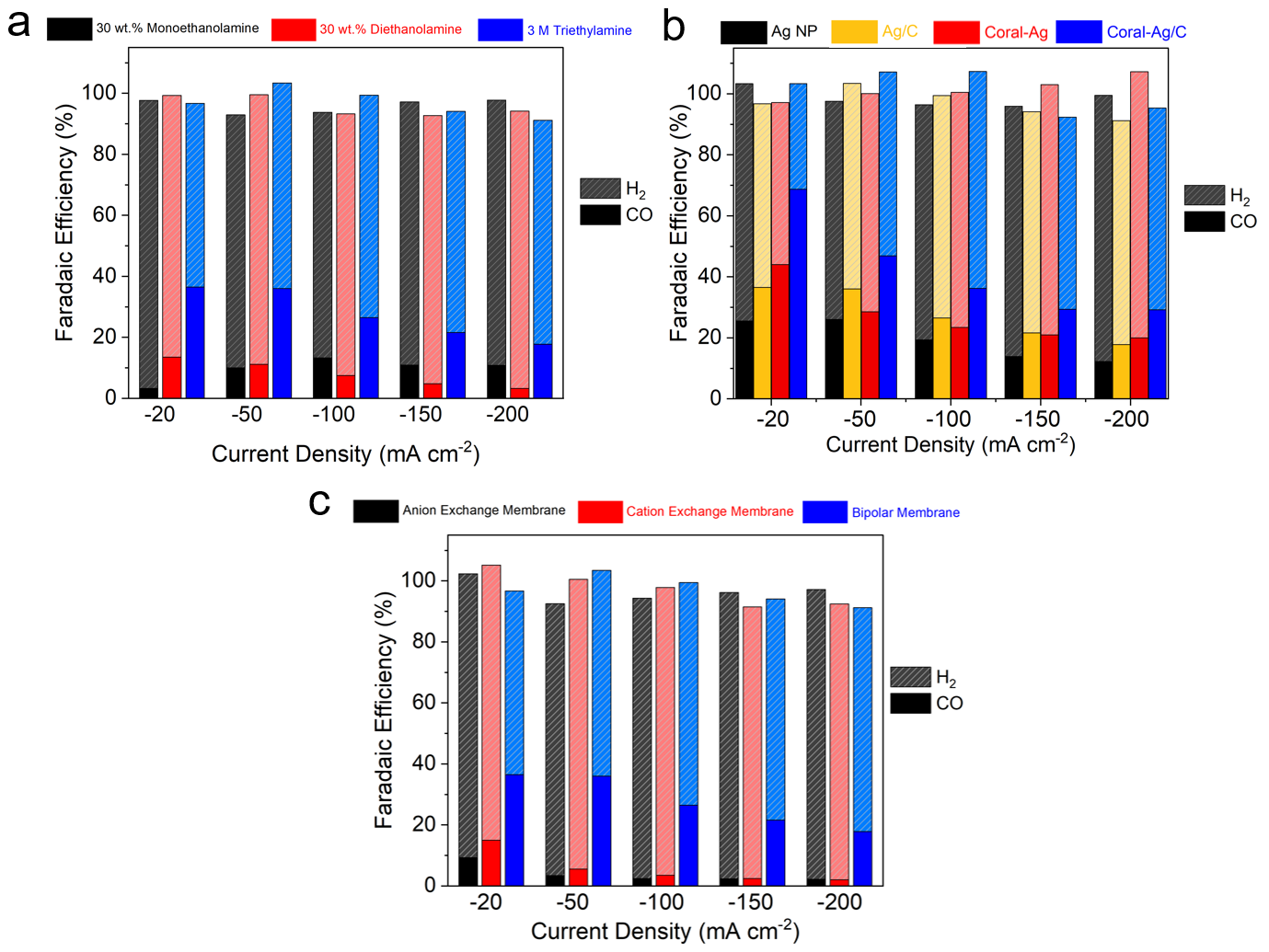


## **Figure S3. CO FE and H_2_ FE for different catalysts with various membranes.** (a) CO FEs (plain) and H_2_ FEs (dash) for Ag/C measured with various applied current densities in monoethanolamine (black), diethanolamine (red), and TREA (blue). (b) CO FEs (plain) and H_2_ FEs (dash) for Ag NP (black), Ag/C (yellow), coral-Ag (red), and coral-Ag/C (blue) in 3 M TREA. (c) CO FEs (plain) and H_2_ FEs (dash) for Ag/C measured with various membranes, including an anion exchange membrane (black), cation exchange membrane (red) and bipolar membrane (blue), in 3 M TREA.

O_2_

CO

H_2_

CO_2_


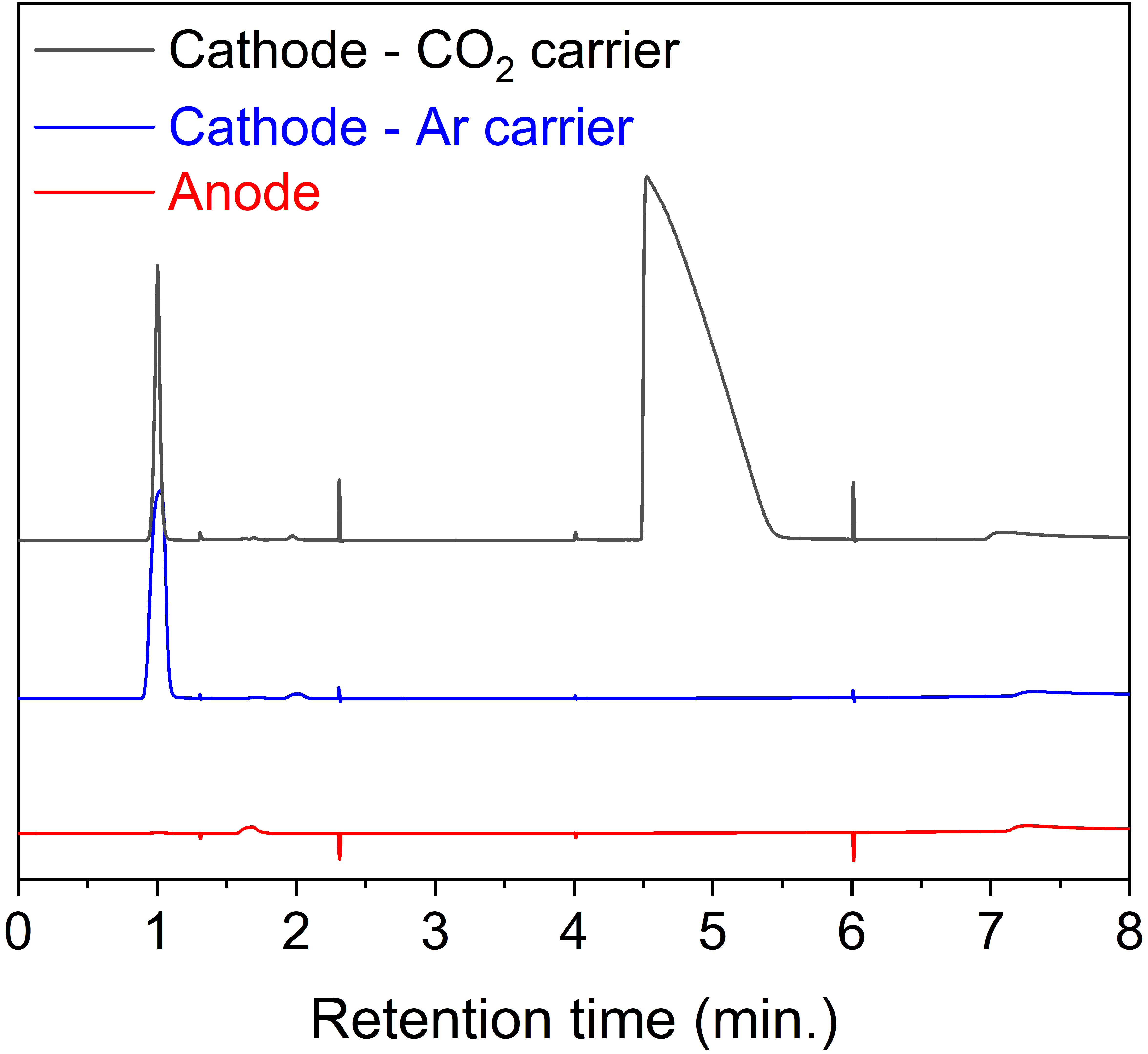


## **Figure S4. GC chromatogram of gas samples under various conditions.** Gas products from eCO_2_R when CO_2_ gas (black) and Ar gas (blue) are respectively used as a gas carrier for GC analysis. Gas products from anode during eCO_2_R with Ar gas carrier (red). The meaning of each peak is shown in the graph.

**
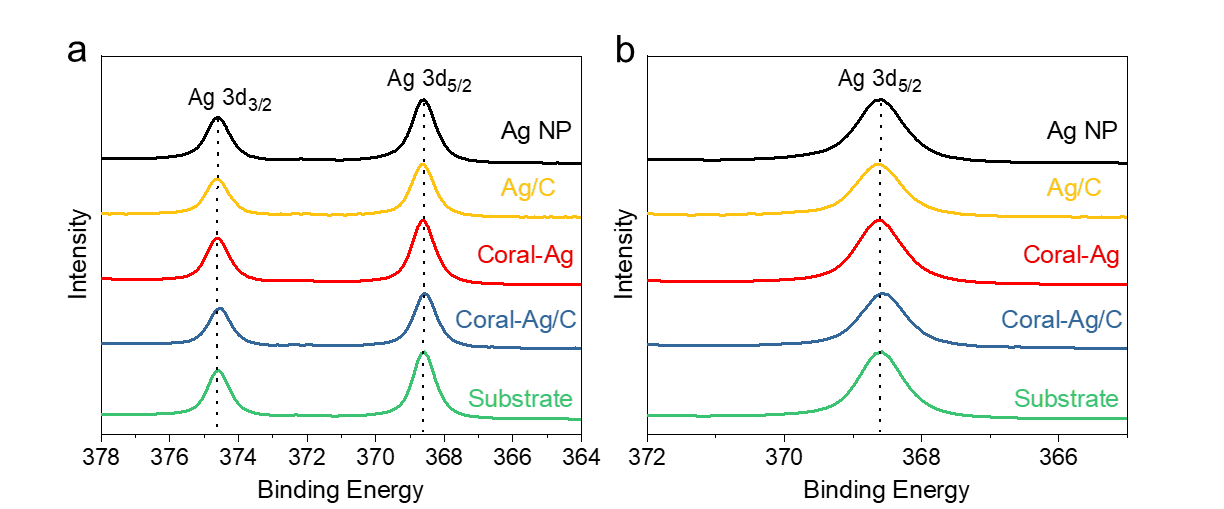
**

## **Figure S5. Chemical state of the prepared Ag catalysts.** (a) XPS-Ag 3d spectra of prepared Ag NP (black), Ag/C (yellow), Coral-Ag (red), Coral-Ag/C (blue) and substrate (green) electrodes. (b) Comparison of Ag 3d_5/2_ peaks position of prepared Ag electrodes.

## **Figure S6. XRD spectra of the prepared Ag catalysts.** Ag NP (black), Ag/C (yellow), Coral-Ag (red), Coral-Ag/C (blue) and substrate (green) electrodes. Star symbol for the peak of carbon. All emerged peaks corresponding to (111), (200), (220), (311) and (222) planes of Ag.

**
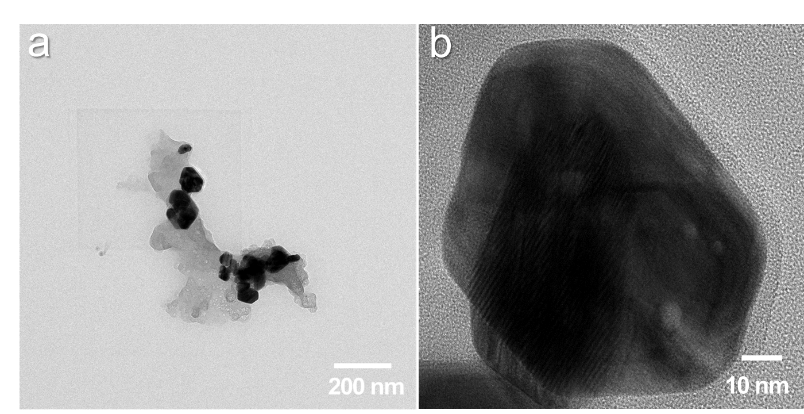
**

## **Figure S7. Morphology of coral-Ag/C.** (a) TEM and (b) HR-TEM image of coral-Ag/C.


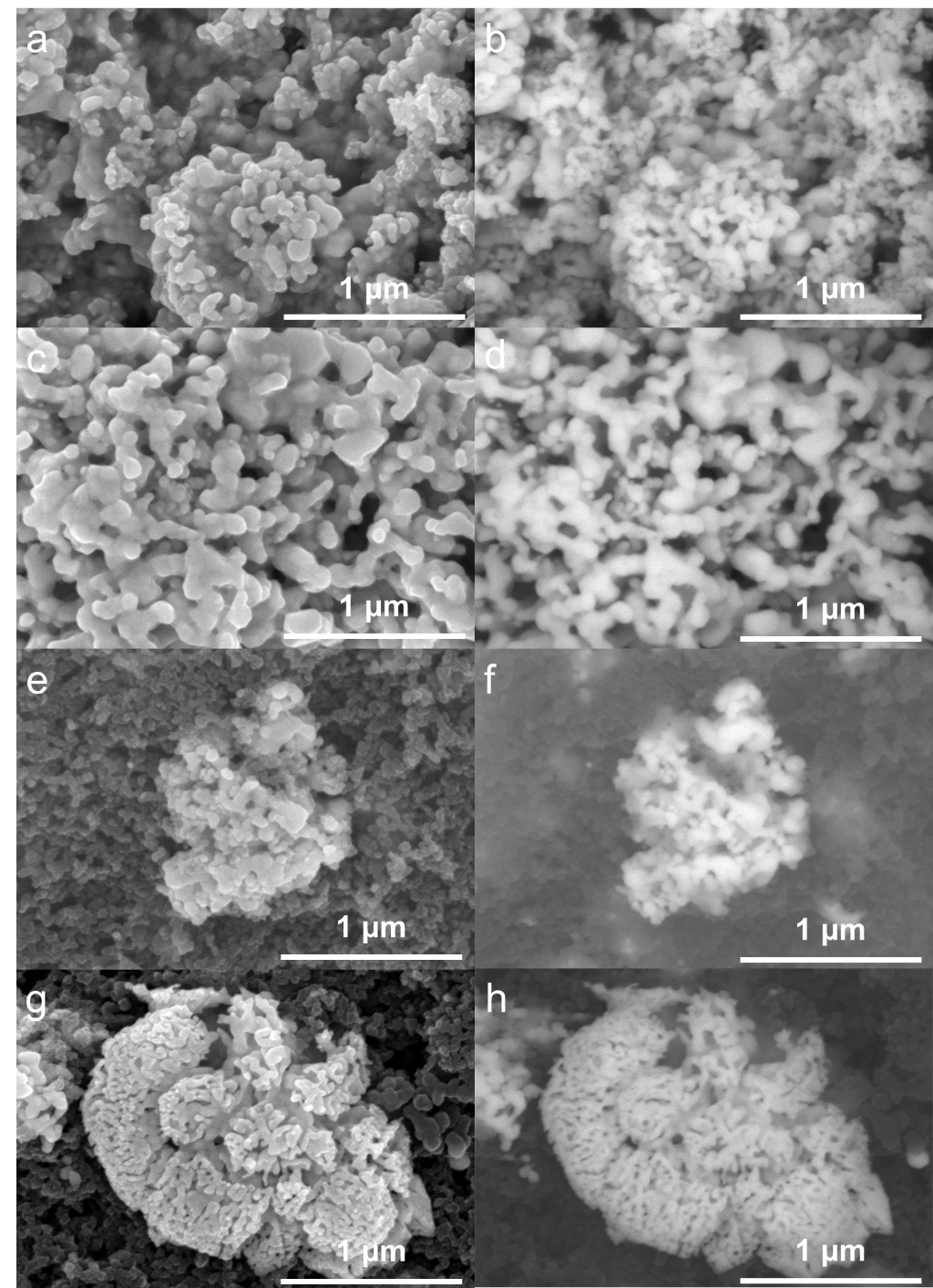


## **Figure S8. Structure of the prepared Ag catalysts.** (a) SEM and (b) BSE images of Ag NP, (c) SEM and (d) BSE images of coral-Ag (e) SEM and (f) BSE images of Ag/C, (g) SEM and (h) BSE images of coral-Ag/C.

## **Figure S9. Applied potential for eCO_2_R.** The applied potential for -20 mA cm^-2^ to -200 mA cm^-2^ of Ag NP (black), Ag/C (yellow), Coral-Ag (red), and Coral-Ag/C (blue) electrode tested during CO_2_RR in 3 M TREA.

## **Figure S10. The eCO_2_R performance of substrate (Ag e-beam).** CO Faradaic efficiency (green bar) and applied potential (green line) of substrate (Ag e-beam) electrode in 3M TREA.


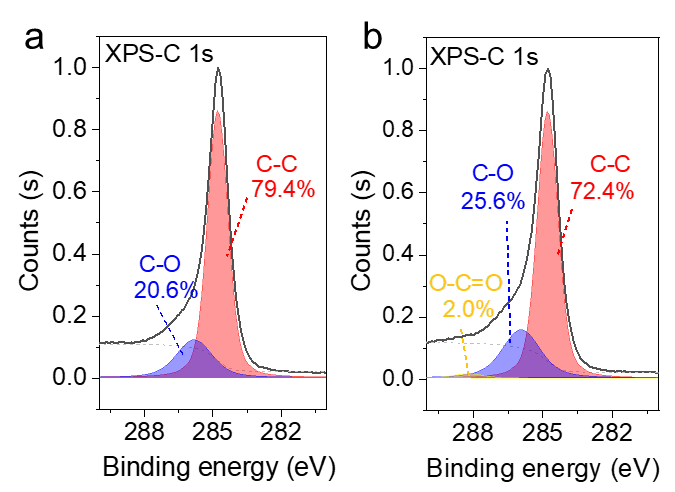


## **Figure S11. Differences in carbon supporter by electrochemical process for coral-structure.** The XPS-C 1s of (a) Ag/C and (b) coral-Ag/C.


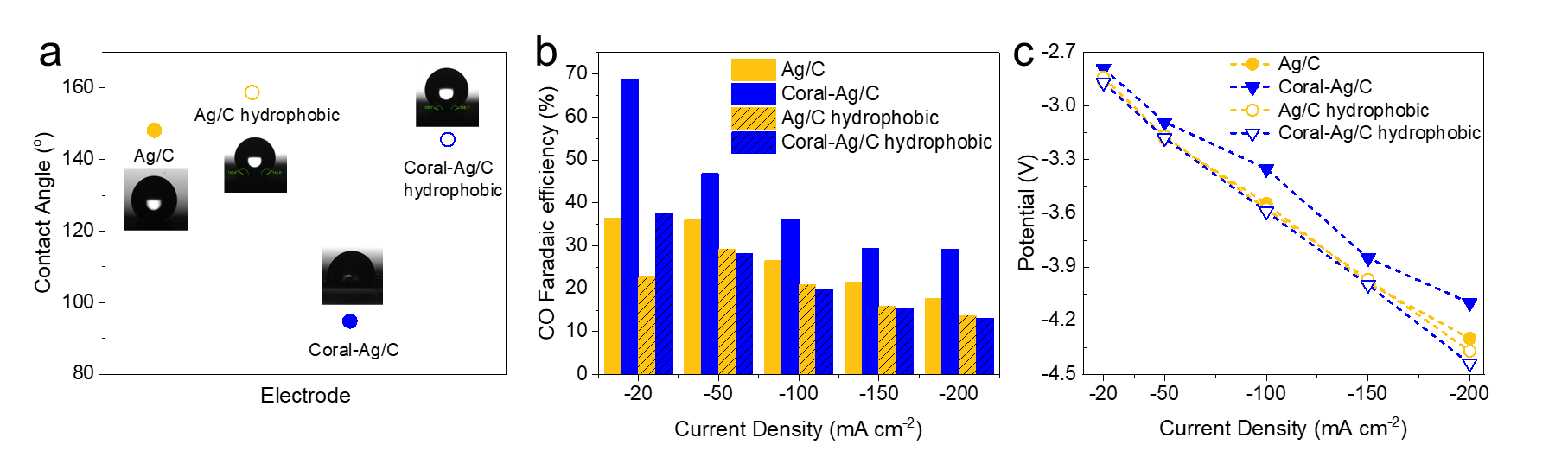


## **Figure S12. Hydrophilic/phobic effect on CO production performance.** (a) Contact angle analysis of prepared hydrophobic Ag/C and hydrophobic coral-Ag/C electrodes compared to Ag/C and coral-Ag/C. (b) CO Faradaic efficiency and (c) applied potentials of prepared hydrophobic electrodes in the RSA system displayed with those of Ag/C and coral-Ag/C.

Note: To prepare the hydrophobic electrodes, the hydrophobic carbon paper (Toray Carbon Paper 120, 5% Wet-Proofed) was used as a substrate while the other processes (i.e., spraying and/or electrochemical process for coral structure) performed the same way. The hydrophobic substrate may interfere with efficient contact between electrodes and electrolytes and contribute to low performance during eCO_2_R, even though the same catalysts is used.

## **Supplementary Note 4: Comparison of CCU processes through modeling approach**

**4.1. Description of three CCU processes**

The purpose of modeling in this study is to find an economically and environmentally feasible CCU technology in order for the replacement of fossil fuel-based syngas production. The three CCU processes considered in this study are ⅰ) thermal CO_2_ conversion (RWGS, reverse water gas shift reaction), ⅱ) electrochemical CO_2_ conversion (gas eCO_2_R), and ⅲ) electrochemical bicarbonate conversion (RSA, reaction swing absorption) for syngas production. A few other technologies, such as solid oxide electrolysis cell (SOEC) and dry methane reforming, can be options for CO_2_ utilizing syngas production. In particular, SOEC is one of the promising alternatives producing CO_2_ oriented syngas because of its low cell potential and relatively high technological maturity [1]. Recently, large scale demonstrations for SOEC are in progress by Sunfire and Haldor-Topsoe. Although immediate problems, such as high temperature durability, must be solved for commercialization, SOEC needs to be assessed as a realistic solution for CO_2_ conversion syngas production in following studies.

Flowsheets of these processes are illustrated in Fig. S13. Both the RWGS and gas eCO_2_R processes require not only a stripping column to regenerate amine but also a pressure swing adsorption (PSA) system to separate unreacted CO_2_ and products (CO and H_2_). Also, the reactor and PSA system are operated at different temperature in both the RWGS and gas eCO_2_R processes so heat integration by heat exchanger network is needed. On the other hand, the RSA process uses neither the regeneration column nor PSA system, because unreacted CO_2_ is still in the amine solution while low soluble products are in gaseous phase. Moreover, the RSA process has no need for heat integration, as it is operated at ambient temperature. The RWGS process produces CO only, so an on-site water electrolysis system (not in the flowsheet) is assumed to supply H_2_ feed. Although the CO_2_ and bicarbonate electrolyzers can produce H_2_ as a by-product, the on-site water split system is also applied for the gas eCO_2_R and RSA processes in order to satisfy H_2_/CO ratio of 2 (desirable syngas for methanol) in the product stream.

The process models are based on the literature as well as our experiment. The RWGS process is modeled, based on the result of the stable 80-hour operation of RWGS reaction from Sun et al. [2]. Cell voltage, current density, and Faradaic efficiency (FE) of the gas eCO_2_R are obtained from the experimental study of Liu, et al. [3] for over 3,000 hours. The PSA system for the RWGS and gas eCO_2_R processes is operated at 300℃ [4]. Energy consumption, capital investment, and operating cost of the PSA system are calculated by parameters from Jouny et al. [5]. When H_2_ is produced by water electrolysis, a polymer electrolyte membrane (PEM) system is assumed to be constructed on the syngas production site [6]. The experiment data of bicarbonate electrolysis and chemisorption by TREA are used for the RSA process modeling. The relations among cell voltage, FE, and current density in the bicarbonate electrolyzer are shown in Fig. S14.


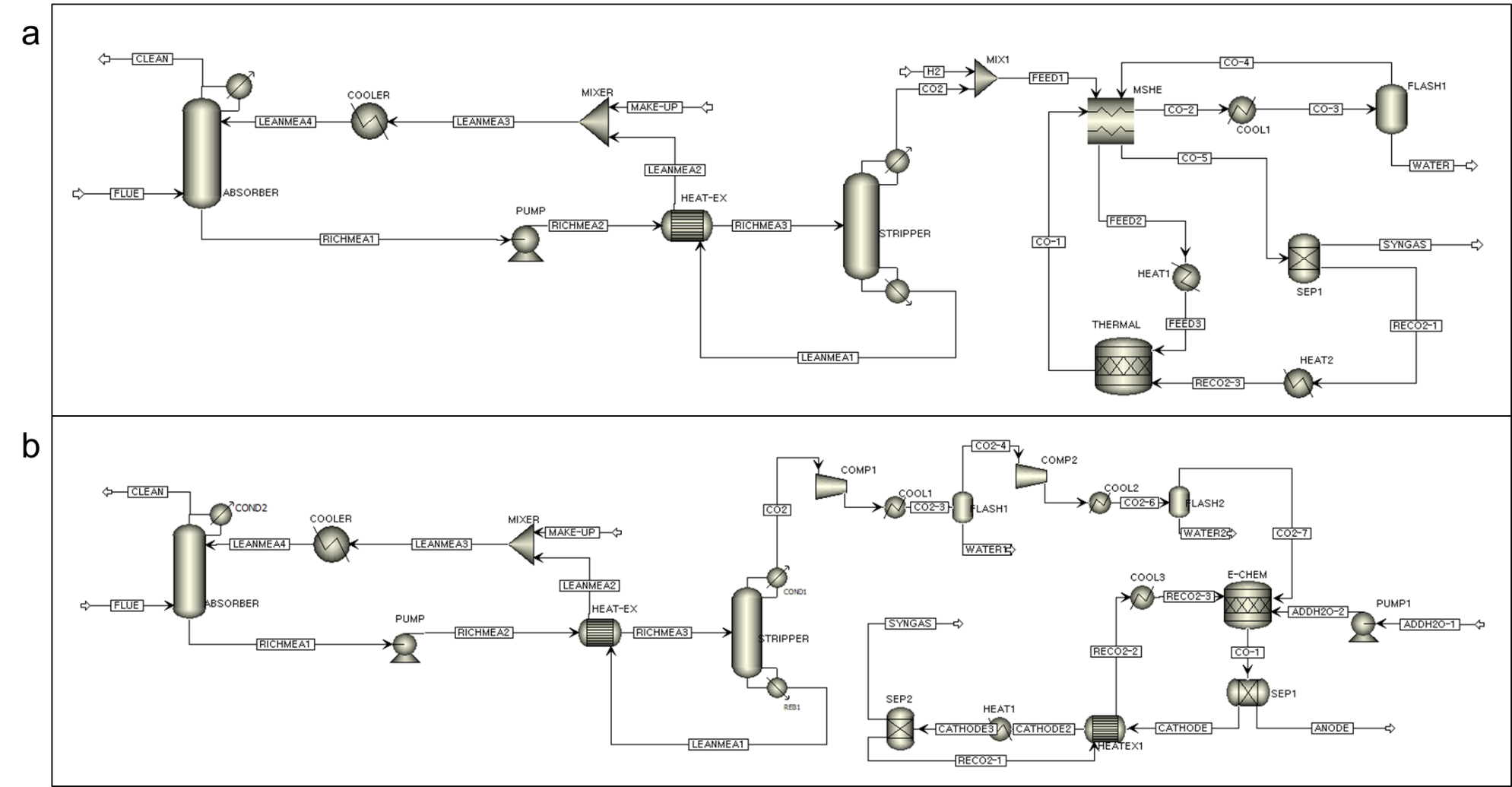


**Figure S13.** **Flowsheets of CO_2_ conversion processes.** (a) RWGS process. (b) Gas eCO_2_R process. (c) CO_2_ RSA process (cont.)


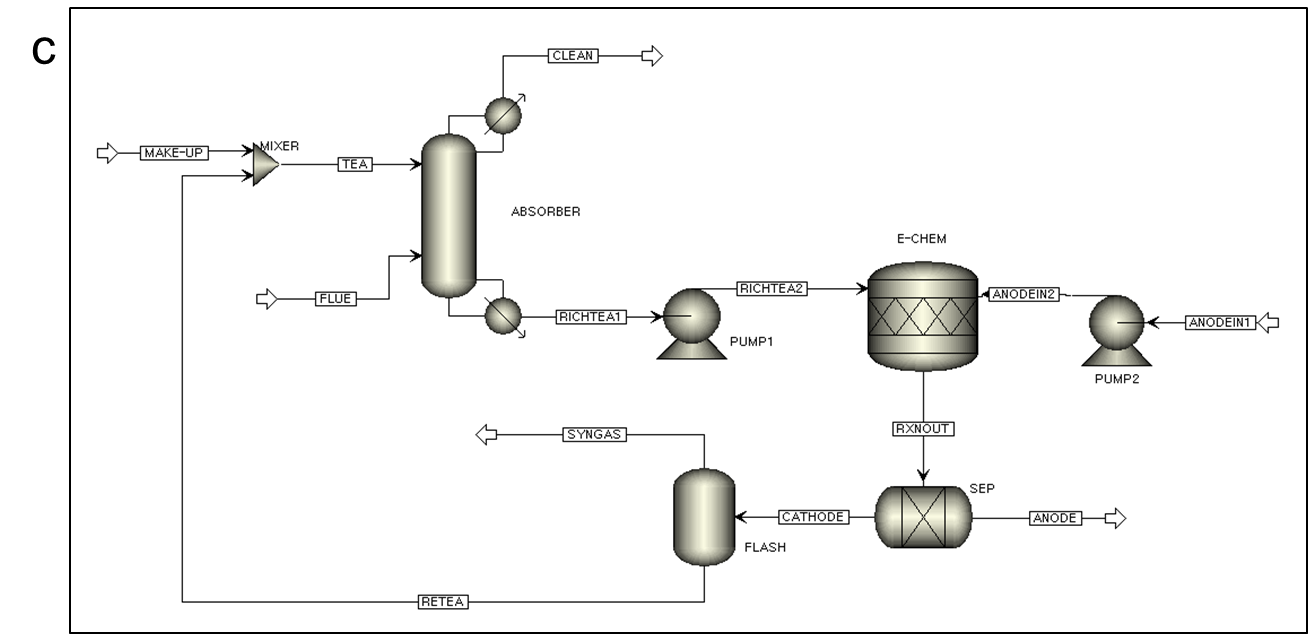


## **Figure S13. Flowsheets of CO_2_ conversion processes.** (a) RWGS process. (b) Gas eCO_2_R process. (c) CO_2_ RSA process.


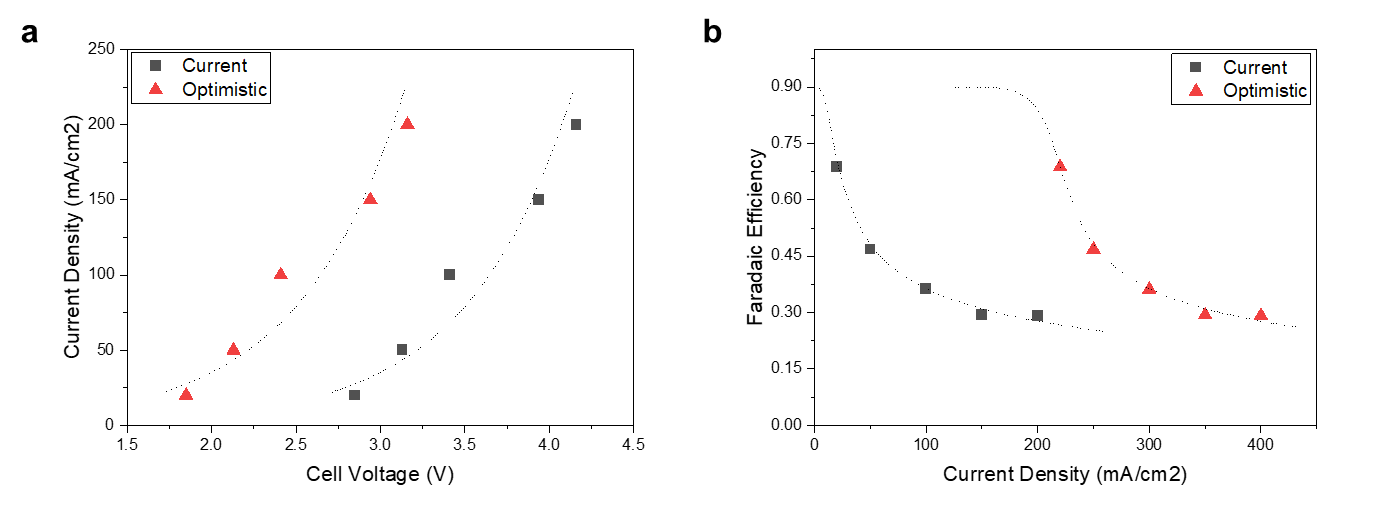


## **Figure S14. Experiment data and future expectation of the bicarbonate electrolysis performance.** (a) Current density curves of current and optimistic scenarios. (b) Faradaic efficiency curves of current and optimistic scenarios.

**4.2. Model development for techno-economic analysis, life cycle assessment, and global sensitivity analysis**

Three process models in this study are developed on Aspen Plus V11 modeling environment using the assumptions explained in the above section. The flowsheets of the three processes in Aspen Plus are described in Fig. S13. The non-random two-liquid model (ELECNRTL) is used as a physical property package. Most of the materials used for modeling (CO_2_, CO, H_2_, H_2_O, N_2_, O_2_, MEA, HCO_3_^-^, CO_3_^2-^, H_3_O^+^, OH^-^, MEAH^+^, and MEACOO^-^) are in the embedded databanks in Aspen Plus. TREA and TREAH^+^ are not in the databanks so added by user-defined molecular structures. Chemisorption kinetics are obtained from the experiment result of the absorption column in Fig. S2. The design basis can be seen in Table S2.

## **Table S2. Design basis for process simulations.**

|  | **RWGS** | **eCO_2_R** | **RSA** |
| --- | --- | --- | --- |
| **Flue Gas Flowrate (kg/h)** | 67,500 | 67,500 | 67,500 |
| **Flue Gas Composition (mol%)** |  |  |  |
| N_2_ | 75.0 | 75.0 | 75.0 |
| O_2_ | 4.0 | 4.0 | 4.0 |
| CO_2_ | 14.0 | 14.0 | 14.0 |
| H_2_O | 7.0 | 7.0 | 7.0 |
| **Feed Temperature (℃)** | 40 | 40 | 40 |
| **Feed Pressure (bar)** | 1 | 1 | 1 |
| **Absorber Column** |  |  |  |
| Diameter (m) | 5 | 5 | 7.5 |
| Height (m) | 17.5 | 17.5 | 22.5 |
| Pressure (bar) | 1 | 1 | 1 |
| CO_2_ Capture Rate (%) | 97.1 | 97.1 | 97.1 |
| Lean Amine (mol%) | 6.3 | 6.4 | 8.7 |
| **Regeneration Column** |  |  |  |
| Diameter (m) | 5 | 5 | - |
| Height (m) | 14 | 14 | - |
| Pressure (bar) | 1.36 | 1.36 | - |
| CO_2_ Purity (%) | 90.0 | 90.0 | - |
| **Reactor/Electrolyzer** |  |  |  |
| Temperature (℃) | 750 | 40 | 53 |
| Pressure (bar) | 1 | 30 | 50 |
| CO_2_ Conversion (%) | 49.7 | 15.0 | 99.0 |
| Cell Voltage (V) | - | 3.0 | Optimized |
| Faradaic Efficiency (%) | - | 90.0 | Optimized |
| Current Density (mA cm^-2^) | - | 200 | Optimized |
| **PSA Temperature (℃)** | 300 | 300 | - |
| **Cooler Outlet Temperature (℃)** | 40 | 40 | 40 |

The chemisorptions in the absorption and desorption columns contain ions. The common reactions for all three processes are auto-ionization of water, ionization of carbonic acid, and CO_2_ release from bicarbonate:

$2H_{2}O\to H_{3}O^{+}+OH^{-}$ (4)

$\mathrm{HCO}_{3}^{-}+H_{2}O\to\mathrm{CO}_{3}^{-}+H_{3}O^{+}$ (5)

$\mathrm{HCO}_{3}^{-}\to\mathrm{CO}_{2}+\mathrm{OH}^{-}$ (6)

The amine-related chemisorption reactions have differences between MEA and TREA, as TREA consumes CO_2_ for bicarbonate whereas MEA produces carbamate:

$MEA+\mathrm{CO}_{2}+H_{2}O\to MEACOO^{-}+H_{3}O^{+}$ (7)

$MEA+H_{3}O^{+}\to MEAH^{+}+H_{2}O$ (8)

$TREA+\mathrm{CO}_{2}+H_{2}O\to TREAH^{+}+HCO_{3}^{-}$ (9)

$TREA+H_{3}O^{+}\to TREAH^{+}+H_{3}O^{+}$ (10)

The reactions in the electrolyzers and RWGS reactor are modeled in the below equations. The extents or conversion rates of those reactions are based on experimental results, studies in the literature, and operating conditions, such as voltage.

$\mathrm{CO}_{2}\to CO+\frac{1}{2}O_{2} for electrolyzers$ (11)

$H_{2}O\to H_{2}+\frac{1}{2}O_{2} for electrolyzers$ (12)

$\mathrm{CO}_{2}+H_{2}\to CO+\frac{1}{2}O_{2} for RWGS reactor$ (13)

The two main outputs of economic evaluation are capital investment and operating cost. Matlab software calculates them, based on the process simulation result from Aspen Plus. Therefore, the linkage between Matlab and Aspen Plus is used in order to perform detailed analyses of those processes.

The capital investment calculations for the processes start from equipment costs. Equipment costs are obtained from the literature [7], except electrolyzers and PSA systems that are in other literature [5]. Base cost (BC) of equipment is estimated by reference size or capacity with reference cost factor. Although equipment have same size or capacity, equipment costs are different by various operating conditions. Therefore, Guthrie material and pressure factors (MPF) are applied for uninstalled cost.

$BC= C_{0}{(\frac{S}{S_{0}})}^{\alpha}$ (14)

$Unistalled cost=\left( \mathrm{BC} \right)(MPF)$ (15)

In general, installation cost of equipment is assumed to be proportional to the base cost, BC (not uninstalled cost), so that module factor (MF) is multiplied to the cost.

$Installation cost=(BC)(MF)-BC=(BC)(MF-1)$ (16)

$Bare module cost=\left( \mathrm{BC} \right)\left( \mathrm{MPF} \right)+\left( \mathrm{BC} \right)\left( MF-1 \right)=(BC)(MPF+MF-1)$ (17)

Since the reference cost factor, C_0_, is based on a specific time, equipment cost needs to be updated to recent value. All equipment costs are updated to 2020 by Chemical Engineering Plant Cost Index (CEPCI).

$Updated bare module cost (UBMC)=\left( \mathrm{BC} \right)\left( MPF+MF-1 \right)*\frac{\mathrm{CEPCI}_{2020}}{\mathrm{CEPCI}_{1968}}$ (18)

Capital expenditure (CAPEX) consists of working capital and fixed capital costs, the fixed capital is divided into manufacturing and non-manufacturing cost, and the manufacturing cost is classified by (updated) bare module cost and contingency cost. These costs are calculated, according to the literature [7].

$$CAPEX=Fixed capital+Working capital=Manufacturing cost+Nonmanufacturing cost+Working capital$$

$=UBMC+Contingency cost+Nonmanufacturing cost+Working capital$ (19)

In the case of operating cost, total production cost is calculated, which consists of direct production, fixed charges, plant overhead, administrative, distribution and selling, and research and development costs [8]. Among those elements shown in Fig. S15, direct cost occupies two-third of the total cost as it contains major cost factors, such as materials, labor, utility, maintenance, supply, lab/QC/QA, and royalty costs. Then, break-even price of syngas is determined by finding net present value (NPV) of zero. The parameters used in the techno-economic analysis are listed in Table S3.

## **Table S3. Parameters for techno-economic evaluation.**

| **Parameter** | **Value** |
| --- | --- |
| Plant Life (year) | 20 |
| Interest Rate (%) | 7.0 |
| Taxation (%) | 38.9 |
| Operating Time (day/year) | 330 |
| Labor Rate ($/[hour∙personnel]) | 40 |
| Depreciation Method | Modified Accelerated Cost Recovery System for 10-year property |
| MEA Price ($/kg) | 3.0 |
| TREA Price ($/kg) | 15.0 |
| Process Water Price ($/1000 gal) | 0.8 |
| O2 Price ($/kg) | 0.0 |
| Electricity Price ($/kWh) | Case Dependent |
| Natural Gas Fuel Price ($/SCF) | 5.0e-3 |
| Steam Price ($/kg) | 0.014 |
| Cooling Water Price ($/kg) | 2.9e-5 |
| On-site H_2_ Production by Water Electrolysis |  |
| Electricity Consumption (kWh/kg) | 54.3 |
| Additional Cost ($/kg) | 1.74 |


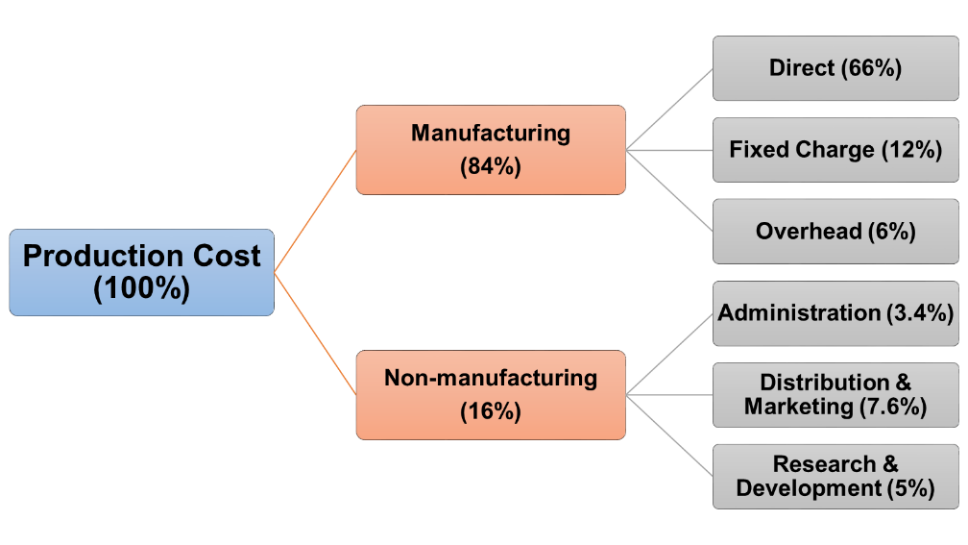


## **Figure S15. Components and their proportion on total production cost.**

LCAs with impact parameter values of materials, energies, etc. obtained from SimaPro V9.3 are conducted by Matlab, based on the process simulation results from Aspen Plus. The ReCiPe 2016(H) is used as a ‘Cradle-to-Gate’ method with the ecoinvent database V3.8 for the system boundaries in Fig. 4(c). For simplicity, transportation of raw materials, such as amines, to the production site is ignored for LCA. Because the three processes in this study have early technology maturity with different technology readiness levels, there are highly uncertainties and lack of data availability [9–10]. Many LCA studies of CCU technology in the literature have been conducted for comparative assessment using ‘Cradle-to-Gate’ approach with functional unit of mass [11]. As a result, this study employs same methodology and conducts sensitivity analysis and scenario analysis.

The global sensitivity analysis (GSA) is conducted for the RSA process via the data flow in Fig. S16. Total 5,000 samples are generated in Matlab by the Sobol sampling method which is one of quasi-random low-discrepancy sequences. Then, Matlab gives sampling data to Aspen Plus for simulation of the RSA process under the sample condition with arbitrary cell voltage. After the process simulation ends, Matlab takes the simulation result from Aspen Plus and starts to calculate economic factors as well as environment factors. Since the current density (CD) and FE are correlated to cell voltage as in Fig. S14, it is needed for the bicarbonate electrolyzer to determine optimal cell voltage. Consequently, the fminbnd algorithm in Matlab maximizes the break-even price of syngas by adjusting cell voltage and re-simulating the RSA process in Aspen Plus with adjusted CO FE and CD. The optimal results include process operating conditions and economic and environment factors. Overall 5,000 optimization runs are done, and those results are stored in Excel. Then, SobolGSA V3.1 software develops a metalmodel from the optimization result of 5,000 samples, and calculates Sobol indices using the metamodel.


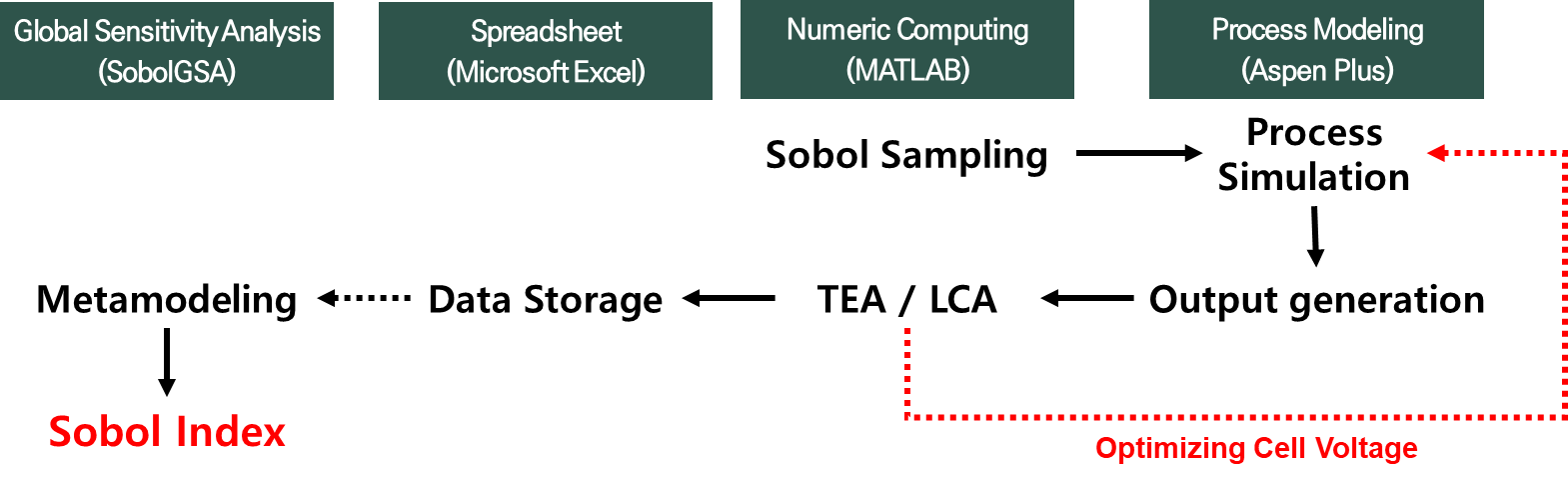


## **Figure S16. Scheme of global sensitivity analysis.**

**4.3. Modeling results**

***4.3.1. Scenarios and cases***

As energy system has been changing from fossil fuel to renewable and the bicarbonate electrolysis is currently at its early development, two scenarios are considered in this study: current and optimistic. In the optimistic scenario, i) the bicarbonate electrolyzer is improved, as seen in Fig. S14, ii) the water electrolysis system for on-site H_2_ production is advanced [6], and iii) electricity generation cost is reduced [12]. Also, there are three cases in each scenario, based on energy sources for electricity generation: energy mix, solar, and wind. Thus, total 18 results (2 scenarios * 3cases * 3 processes) can be obtained.

Table S4 lists parameters values in the current and optimistic scenarios. The electrolyzer-related parameters are FE improvement, CD improvement, and unit cost of bicarbonate elctrolyzers. CD improvement means that the cell voltage at the same CD value will be reduced up to 1 V in the optimistic scenario (Fig. S14a). In the case of FE improvement, increasing up to 200 mA cm^-2^ at the same FE value is assumed for the optimistic scenario (Fig. S14b). The unit cost of electrolyzer in the current scenario comes from the study of Jouny et al. [5].

## **Table S4. Parameters values used for global sensitivity analyses.**

|  | **Current** | **Optimistic** |
| --- | --- | --- |
| CO_2_ Capture Rate (%) | 90 | 95 |
| FE Improvement (mA cm^-2^) | 0 | 200 |
| CD Improvement (V) | 0 | 1 |
| Unit Cost of Electrolyzer ($/m^2^) | 919.7 | 300 |
| Increase of Wind Share in Energy Mix (times) | 1 | 4 |
| Increase of Solar Share in Energy Mix (times) | 1 | 7 |
| Electricity Generation Improvement (%) | 0 | 80 |
| Improvement of Additional Cost of External H_2_ (%) | 0 | 100 |

Regarding electricity price, electricity generation cost of each energy source in the USA is used. The median value of levelized cost of energy (LCOE) in the USA is applied [13]. For example, $117.25/MWh and $44/MWh are the medians of LCOE in the USA for coal and solar, respectively. The electricity generation cost is divided into several factors, such as fuel, carbon, operation and maintenance (O&M), decommissioning and waste management costs, etc. Because the electricity generation by renewables is expected to be improved further, the optimistic scenario is assumed to reduce 80% of all cost sectors except O&M cost for solar and wind (Fig. S17). This optimistic solar and wind costs are near $20/MWh, and it is the theoretical case in the CCU data visualization tool developed by the national renewable energy laboratory in the USA [12].

The current energy mix in the USA is 20% renewables (wind, hydro, solar, biomass, and geothermal), 20% nuclear, 19% coal, 40% natural gas and 1% petroleum [14]. Because the available data of electricity generation cost are natural gas, coal, nuclear, hydro, solar, and wind, these 6 energy sources are used for the energy mix case. These sources occupy 97% of the total energy mix in the USA, so normalized to 100%. In the optimistic scenario of the energy mix case, only solar and wind portions are assumed to increase 7 times for solar (16.1%) and 4 times for wind (33.6%), respectively, so that almost half of electricity is supplied by solar and wind. The other energy sources are shrink, based on their portions in the current scenario to make their total energy mix of 50.3%.

## **Figure S17. Current and optimistic electricity generation costs from different energy sources [13].**

The H_2_ production by an on-site water electrolysis plant is based on the report of James et al. [6]. Its cost consists of electricity cost and additional cost (fixed O&M cost, capital cost, etc.). The total cost of them is considered a material cost for H_2_ in the syngas production processes, because this study assumes H_2_ purchase from the water electrolyzer as the internal H_2_ procurement for the syngas production processes. The report [6] provides the amount of electricity consumption (54.3 vs. 50.2 kWh/kg H_2_) and additional cost ($1.74 vs. $0.74/kg H_2_) for current and optimistic scenarios.

The remained parameter for current and optimistic scenarios is CO_2_ capture rate in the absorber. Most studies have used capture rate of 90% as a standard for comparison. Thus, this study uses the variation of CO_2_ capture rate from 90 to 95% to check the effect of higher capture rate on economic and environment aspects by GSA.

***4.3.2.*** ***Techno-economic analysis***

The economic evaluation of the three processes is performed by the aforementioned approach for two scenarios and three cases. Fig. S18 compares the CAPEX calculation results. Unlike the RWGS and gas eCO_2_R processes, the RSA process shows much improved CAPEX in the optimistic scenario and most of the reduction comes from the decrease of electrolyzer cost. As the bicarbonate electrolyzer has better performance in the optimistic scenario, the reactor part (electrolyzer) of CAPEX substantially lowered. The RSA process in the current scenario has also different CAPEX from energy source to energy source, because of different operating conditions by optimization under different electricity prices. When electricity comes from energy mix, the bicarbonate electrolyzer cost becomes higher than the solar and wind cases in order to reduce electricity cost by decreasing cell voltage but increasing electrolyzer area. For the RWGS and gas eCO_2_R processes, the reactor cost is low, but the addition of the PSA system to separate unreacted CO_2_ and syngas product leads to a great amount of capital investment (Equip. (Others) in Fig. S18).

The operating expenditures (OPEXs) for the three processes are shown in Fig. S19. All three processes have similar OPEX value in the current scenario, but the RSA outperforms in the optimistic scenario due to improved CD and CO FE at even lowered cell voltage. In the current scenario, the optimal cell voltage of the bicarbonate electrolyzer is 3.1V for the energy mix case and 3.4V for solar and wind cases, respectively. The extremely high equipment cost of the bicarbonate elctrolyzer is observed in the current scenario because of the low current density performance (42–72 mA cm^-2^). Although higher cell voltage to increase current density can be applied, this leads to low CO FE (41–51%) and high H_2_ FE (49–59%). It means much of H_2_ in the syngas comes from the bicarbonate electrolyzer at higher voltage than that in the on-site water electrolzer. However, as the bicarbonate electrolysis technology will become mature in the future, approximately 3-fold higher current density with 90% CO FE at 0.3–0.4V lowered cell voltage is expected in the optimistic scenario, making substantial reduction of both CAPEX and OPEX. When the RSA process uses only renewable energy for electricity, syngas price in the optimistic scenario is $0.56/kg syngas for solar and $0.65/kg syngas for wind, respectively. These values can economically compete with the fossil fuel-based conventional technology.


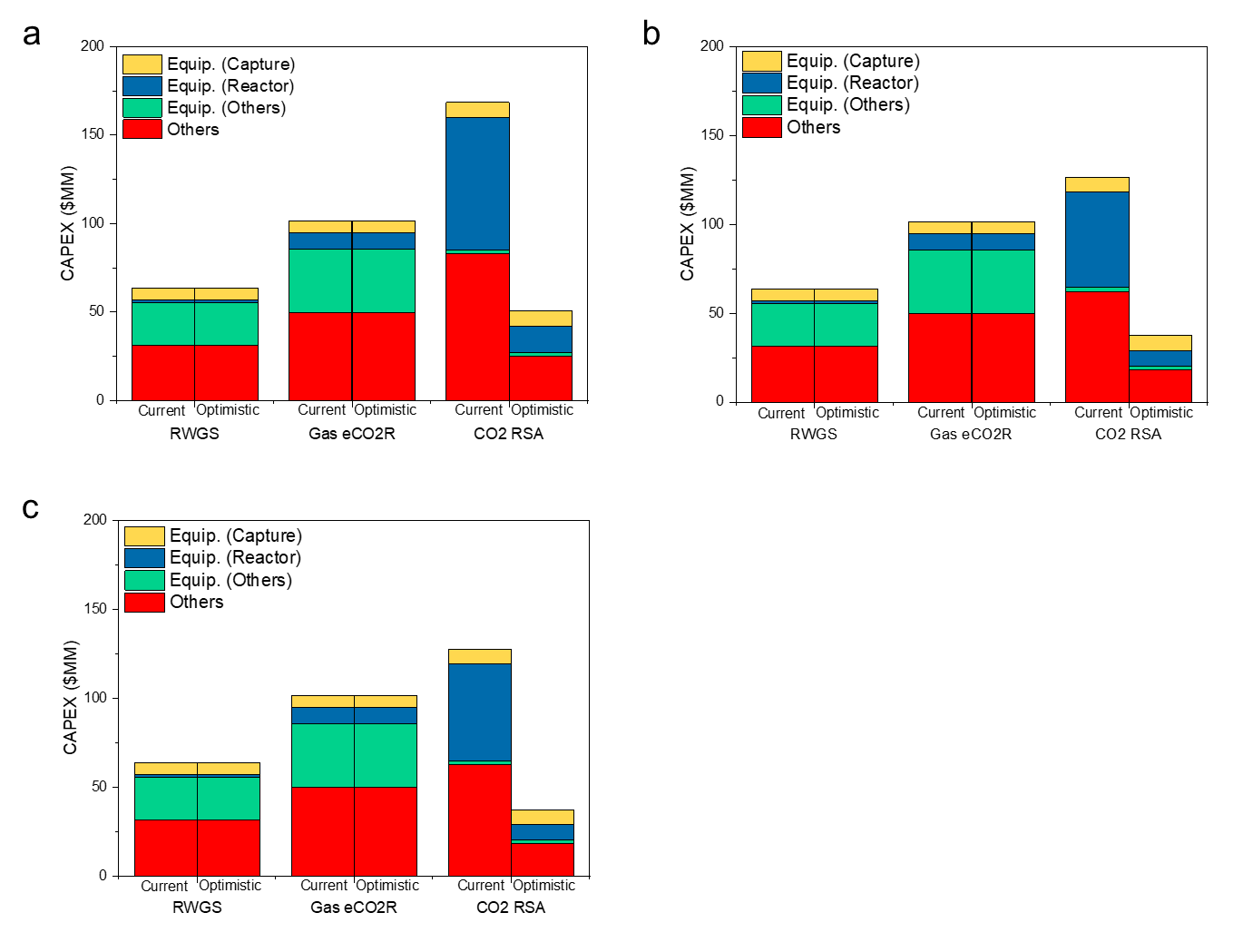


## **Figure S18. Capital investment of three processes under various energy sources.** (a) Energy mix case. (b) Solar case. (c) Wind case.


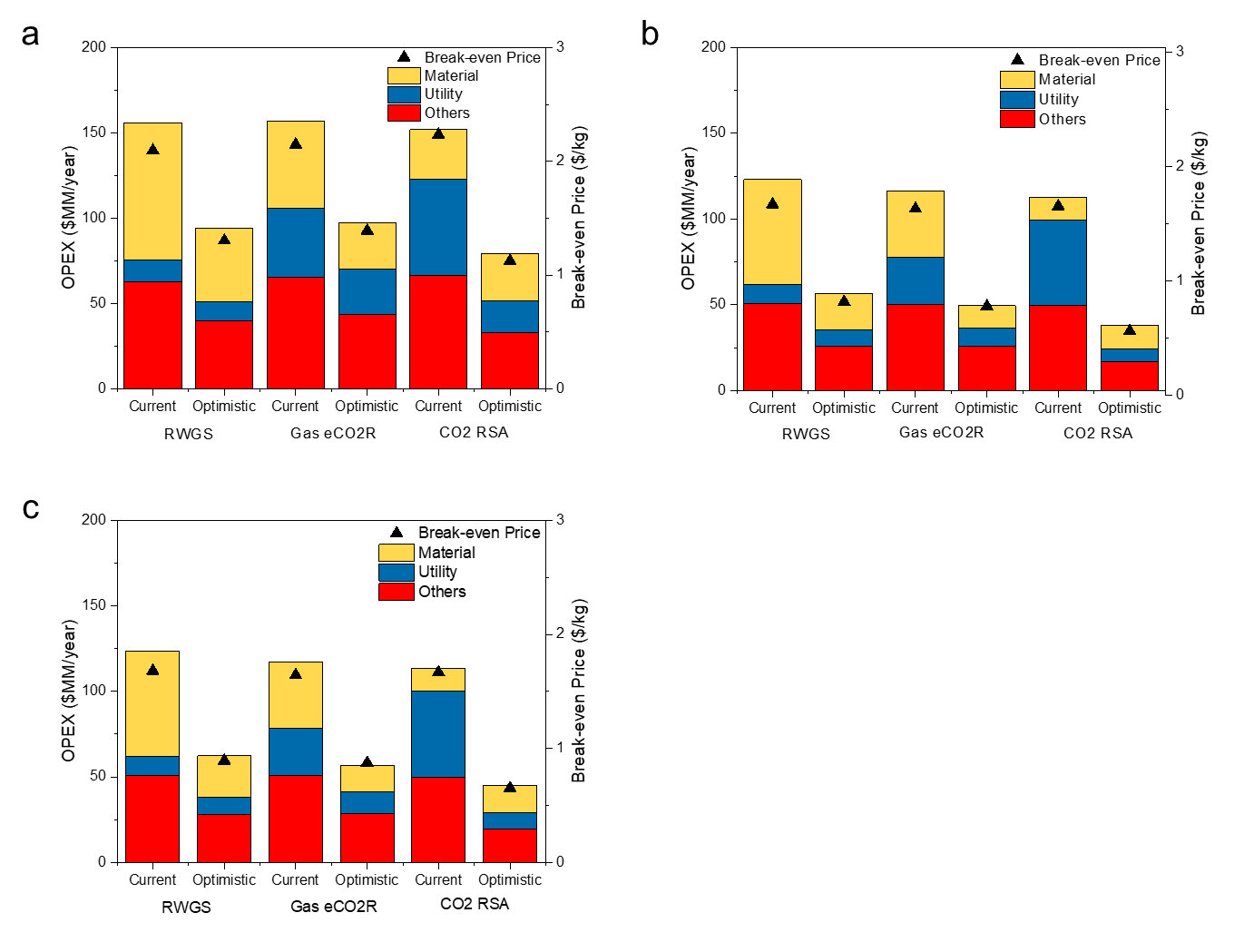


## **Figure S19. Operating cost and break-even price of three processes under various energy sources.** (a) Energy mix case. (b) Solar case. (c) Wind case.

***4.3.3. Life cycle assessment***

The LCA result is shown in Fig. S20. The result is considerably sensitive to energy source, because the three processes are highly energy intensive and CO_2_ emission is extremely different from energy source to energy source. For instance, greenhouse gas emissions of electricity are 820, 490, 41 and 12 g CO_2_/kWh for coal, gas, solar and wind, respectively [5].

The main CO_2_ emission in the RWGS process is related to the material sector, and most of it comes from the purchased H_2_ which is produced by the on-site water electrolysis. If energy source for electricity is changed to renewables, the emission for raw materials production can be significantly reduced. However, the emission from the utility sector is decreased little, as heating by natural gas for thermal synthesis is the major usage of utilities. In the case of the gas eCO_2_R process, CO_2_ emissions from both materials and utility are simultaneously decreased when changing energy sources to renewables. This is because the on-site H_2_ production (material sector) and CO_2_ conversion (utility sector) consume a large amount of electricity. The RSA process consumes a large amount of electricity for CO_2_ conversion with by-product H_2_ in the bicarbonate electrolyzer. Thus, utility sector is the major sector for CO_2_ emission in the RSA. For the fossil resource scarcity, the similar trend to the GWP can be seen for all three processes.

As a result, the RSA process with the wind case in the optimistic scenario seems the best selection of syngas production from the economic and environment points of view: $0.65/kg syngas with CO_2_ emission of 0.27kg CO_2_ eq./kg syngas. When solar energy is used, the break-even price can be lowered to $0.56/kg syngas. However, this is much less environmentally friendly (0.72kg CO_2_ eq./kg syngas) than the wind case.

To provide an overview for the current development level of CCU technologies, the results of techno-economic analyses and LCA of syngas-based chemical production via CCU from the recent literature [15–28] are presented in Fig. S21. To compensate for the differences in cost calculation bases of the papers, i.e., production capacity, currency, and date of evaluation, a relative cost index (RCI), which is calculated by dividing the CCU-based chemical cost by the conventional chemical market cost, is used for evaluation. Note that global warming potential (GWP) is presented as kg CO_2_ eq. per functional unit (FU). While the developed CO_2_ utilization pathways successfully mitigate GWPs, none of the proposed CCU systems is economically competitive with conventional production pathways. Taking into account market margins, it is evident that CCU-based chemicals cannot replace conventional production pathways at the current stage of development, despite the possibility of reducing GWP. It should be noted that the GWP of CCU-based chemical production is more dependent on the energy input source than the method of material conversion. Thus, to develop a viable CCU solution, pathways capable of lowering production costs and integrating larger proportions of renewable energy should be the focus of development.

In Fig. S21, the RCI and GWP of the different studies are evaluated based on the following criteria: first, the $C_{\mathrm{Product}_{\mathrm{CCU}}}$ value is determined as the base case cost value provided by the specific literature. Most studies provide cost values for various scenarios, such as changes in renewable electricity costs, and policy changes such as carbon credits. These changes can greatly alter the $C_{\mathrm{Product}_{\mathrm{CCU}}}$ values, but inherit large uncertainties in terms of the implementation period, or the level of implementation. To allow a fair comparison, the base case of the proposed process/system, which is based on a realistic scenario, is taken as the $C_{\mathrm{Product}_{\mathrm{CCU}}}$ value of that study. Secondly, the GWP value is calculated differently according to the range of the system boundary designated within the study. When a full system boundary is implemented, where the CO_2_ source is included along with the CCU process, the GWP values are calculated by subtracting the conventional GWP value from the GWP of the proposed process. For a restricted system boundary, where only the proposed process is selected as the system of analysis, neglecting the CO_2_ source, the GWP value provided within the study is used. Thirdly, all of the GWP values provided within the references are regarded as GWP100 values, which indicates the GWP within a 100-year period. While some studies state the use of GWP100 during LCA, most of the studies do not specify the LCA evaluation method of use or the GWP evaluation period. Since the most widely used LCA evaluation method is the ReCiPe 2016 Hierarchy method, which evaluates the GWP100 of various chemicals, it is assumed that all of the studies are evaluated based on this method. It should be noted that the considered list of references is non-exhaustive, and certain studies conducting techno-economic analysis can show profitability according to the different assumptions made.


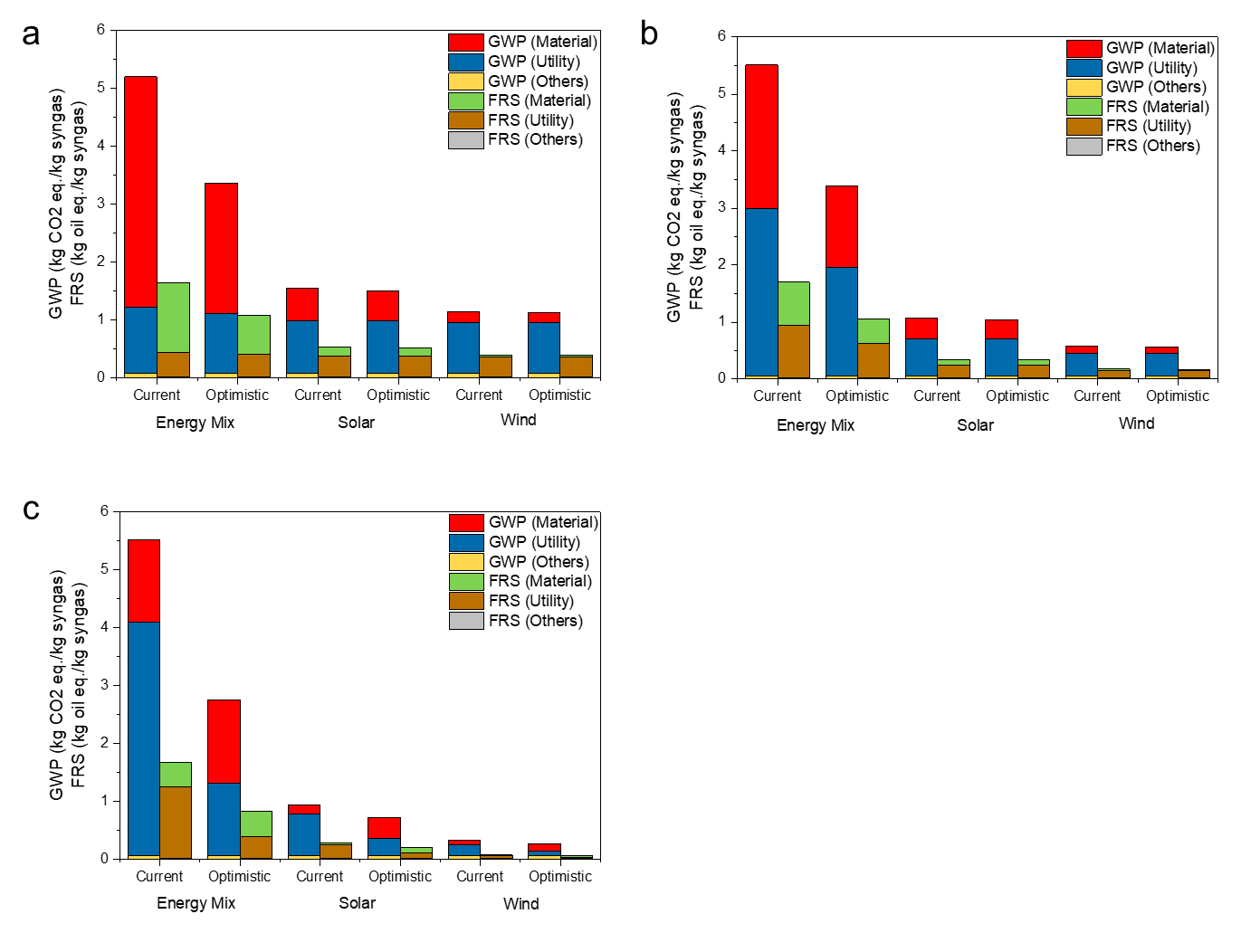


## **Figure S20. LCA result of three processes under various energy sources.** (a) RWGS process. (b) Gas eCO_2_R process. (c) CO_2_ RSA process.


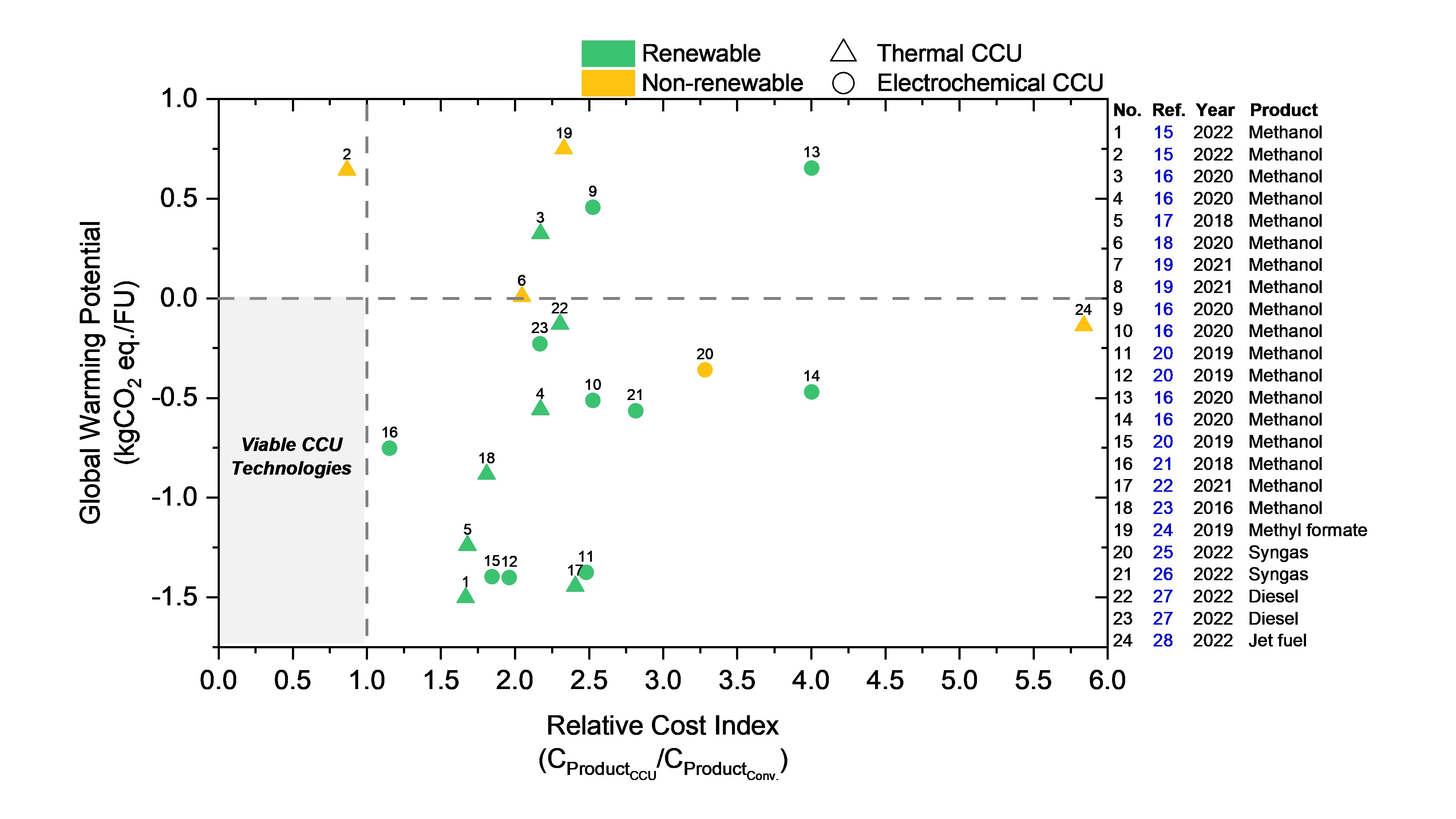


## **Figure S21. RCI and GWP for syngas-based chemical production from CCU pathways in the literature.** While CO_2_ is mitigated in most CCU pathways, none of the pathways are economically competitive compared to conventional chemical production.

***4.3.4. Global sensitivity analysis***

GSAs are carried out for the RSA process under the three cases: energy mix, solar and wind. The uncertain variables (inputs) and their uncertain ranges are listed in Table S4. The GSA can aid decision makings for efficient and swift deployment of the CCU technology by showing sensitivity of uncertain variables and providing the priority of research targets among those variables. The dependent variables (outputs) are related to capital investment, operating cost and environmental impact: CAPEX, material cost, utility cost, OPEX, break-even price of syngas, amount of electricity usage and CO_2_ emission. The 1^st^ and total order Sobol indices for the GSA are illustrated in Fig. S22 for the energy mix case, Fig. S23 for the solar and Fig. S24 for the wind, respectively.

In the energy mix case, the improvement of CO FE, instead of CD, has the highest impact on CAPEX. Whereas increasing current density reduces CAPEX by the smaller bicarbonate electrolyzer area, the higher CO FE decreases CAPEX by the lower H_2_ production rate in the bicarbonate electrolyzer. Therefore, CO FE improvement also affects the materials cost by purchasing more H_2_ from the on-site water electrolysis system and the utility cost by reducing electricity use in the bicarbonate electrolyzer. In the case of the Sobol index of CO_2_ emission, the wind share increase is the most important factor as wind is the least CO_2_ emitting energy source and has the higher variation of its share in energy mix (8.4–33.6%) than that of solar share (2.3–16.1%).

The solar and wind cases have the similar GSA results. The cost improvement of electricity generation by solar and/or wind plays a significant role in the economic and environmental points of view. This is because the utility cost is considerably reduced from the current to the optimistic scenario (Fig. S19) and the renewable energies have a potential for further cost reduction in the future. An interest thing in the wind case is that the CO_2_ capture rate in the absorber column has the highest Sobol index value to the CO_2_ emission. GWP is much lower when wind energy is used (Fig. S20c) as wind is the cleanest energy source. Therefore, the importance of uncaptured CO_2_ in the absorber that is released to the atmosphere is relatively high.


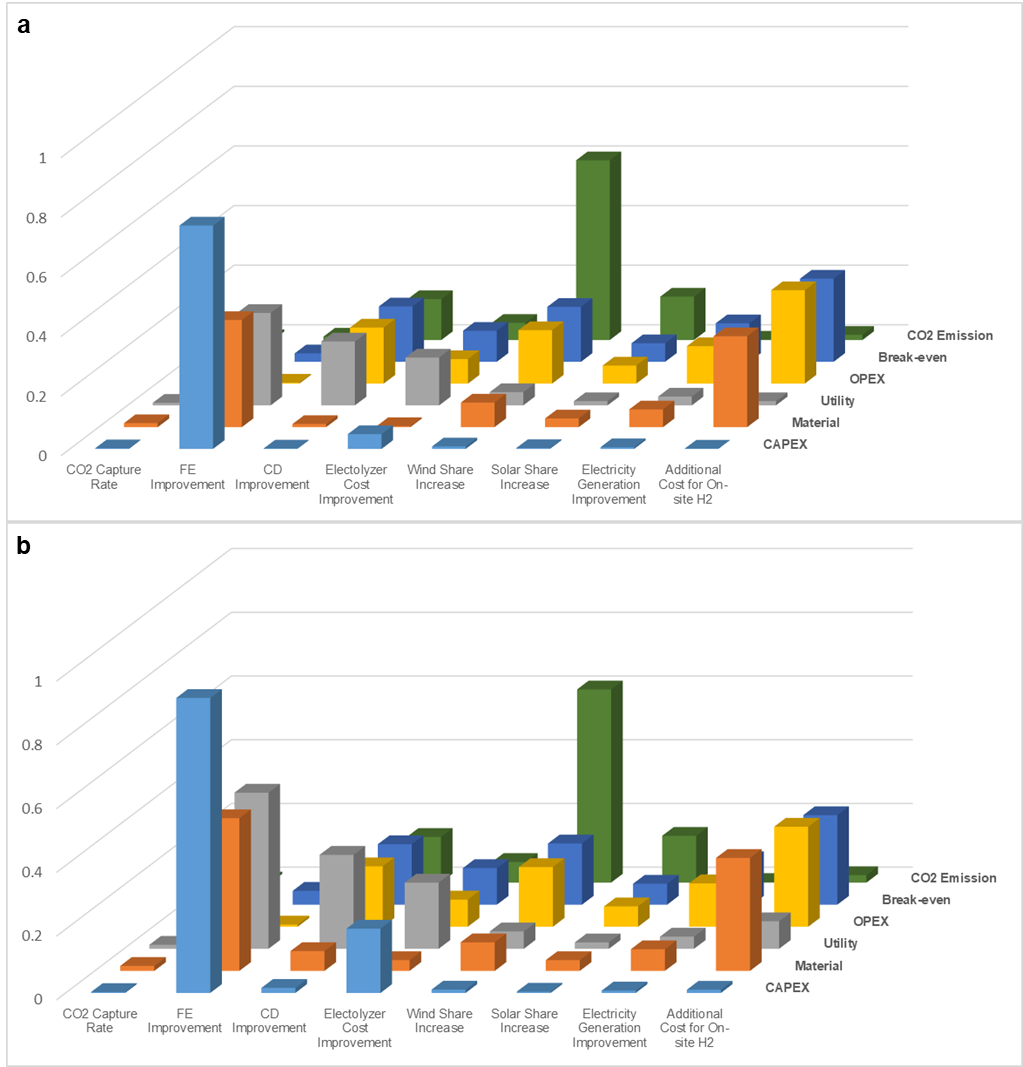


## **Figure S22. GSA result of the RSA process in the energy mix case.** (a) 1^st^ order of Sobol index. (b) Total order of Sobol index.


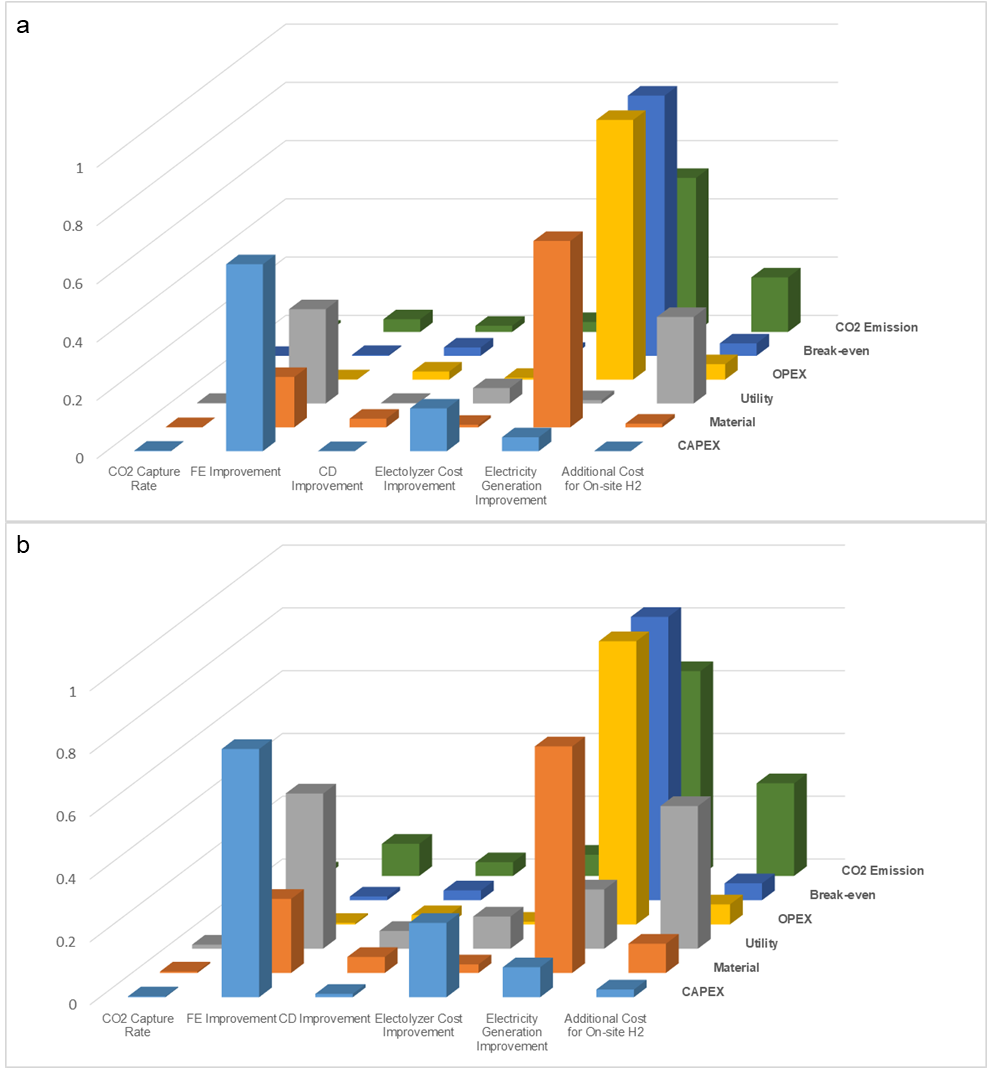


## **Figure S23. GSA result of the RSA process in the solar case.** (a) 1^st^ order of Sobol index. (b) Total order of Sobol index.


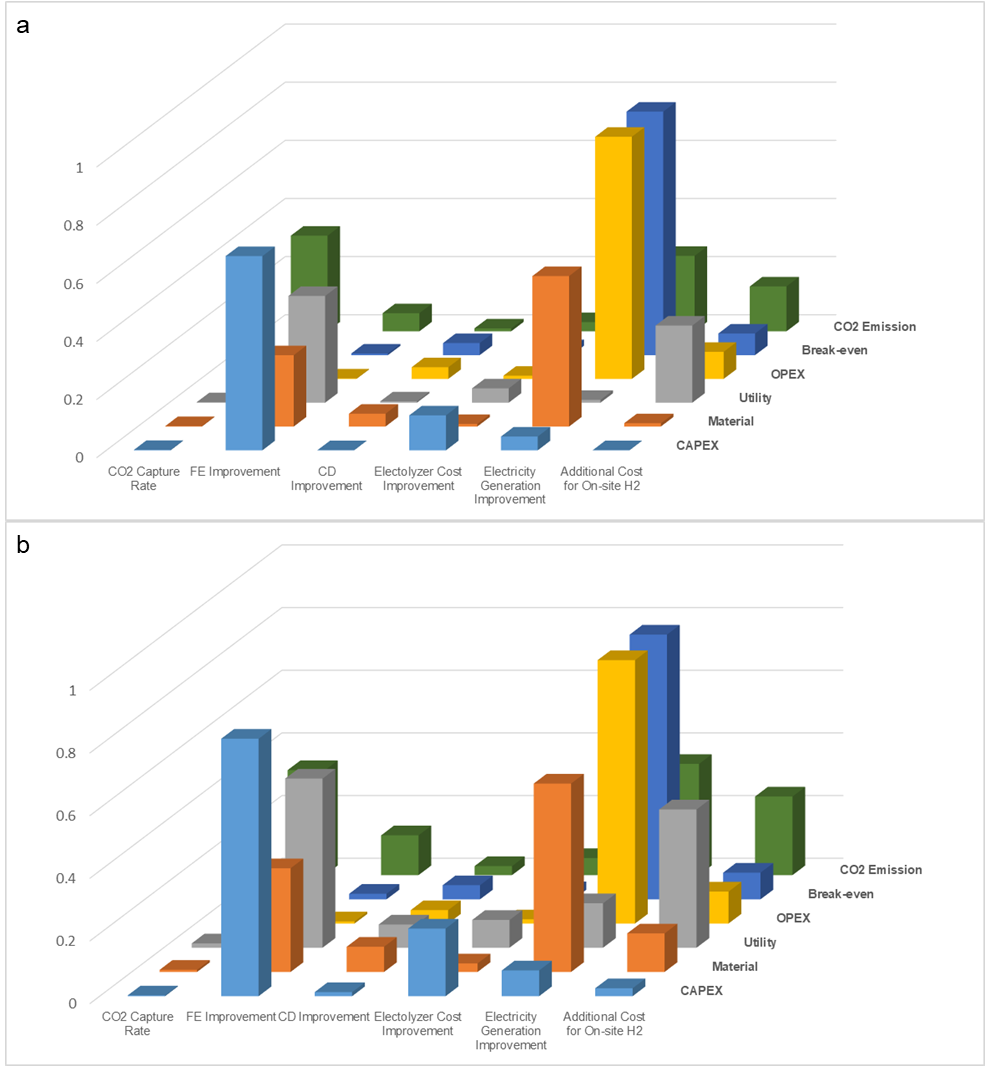


## **Figure S24. GSA result of the RSA process in the wind case.** (a) 1^st^ order of Sobol index. (b) Total order of Sobol index.

**4.4. Reference**

[1] A. Hauch, R. Kungas, P. Blennow, A. B. Hansen, J. B. Hanse, B. V. Mathiesen, M. B. Mogensen; Recent advances in solid oxide cell technology for electrolysis. *Science* (2020), 370, 186.

[2] F. Sun, C. Yan, Z. Wang, C. Guo, S. Huang; Ni/Ce-Zr-O catalyst for high CO_2_ conversion during reverse water gas shift reaction (RWGS). *International Journal of Hydrogen Energy* (2015), 40, 15985-15993.

[3] Z. Liu, H. Yang, R. Kutz, R. I. Masel; CO_2_ Electrolysis to CO and O_2_ at High Selectivity, Stability and Efficiency Using Sustainion Membranes. *Journal of The Electrochemical Society* (2018), 165, J3377

[4] X. Zhu, Y. Shi, S. Li, N. Cai; Elevated temperature pressure swing adsorption process for reactive separation of CO/CO_2_ in H_2_-rich gas. *International Journal of Hydrogen Energy* (2018), 43, 13305-13317.

[5] M. Jouny, W. Luc, F. Jiao; General Techno-Economic Analysis of CO_2_ electrolysis Systems. *Industrial &Engineering Chemistry Research* (2018), 57, 2165-2177.

[6] B. D. James, D. A. DeSantis, G. Saur; Final Report: Hydrogen Production Pathways Cost Analysis (2013-2016). Department of Energy (2016), USA.

[7] L. T. Biegler, I. E. Grossmann, A. W. Westerberg; *Systematic Methods of Chemical Process Design*. Prentice Hall (1997), USA.

[8] M. X. Peters, K. D. Timmerhaus, R. E. West; *Plant Design and Economics for Chemical Engineers (5^th^ edition)*. McGraw-Hill (2002), USA.

[9] L. J. Muller, A. Katelhon, M. Bachmann, A. Zimmermann, A. Sternberg, A. Bardow; A Guideline for Life Cycle Assessment of Carbon Capture and Utilization. *Frontiers in Energy Research* (2020), 8, 15.

[10] A. W. Zimmermann, T. Langhorst, S. Moni, J. A. Schaidle, F. Bensebaa, A. Bardow; Life-Cycle and Techno-Economic Assessment of Early-Stage Carbon Capture and Utilization Technologies – A Discussion of Current Challenges and Best Practices. *Frontiers in Climate* (2022), 4, 841907.

[11] N. Thonemann; Environmental impacts of CO_2_-based chemical production: A systematic literature review and meta-analysis. *Applied Energy* (2020), 263, 114599.

[12] <https://www.nrel.gov/bioenergy/co2-utilization-economics/electrochemical-conversion-pathway.html> [Accessed on March 21, 2022]

[13] International Energy Agency, Nuclear Energy Agency, Organisation for Economic Co-operation and Development; *Projected Costs of Generating Electricity 2020*, IEA (2020)

[14] <https://www.eia.gov/energyexplained/electricity/electricity-in-the-us.php> [Accessed on March 21, 2022]

[15] S. Cho, C. Kim, J. Kim; Techno-economic assessment and early-stage screening of CO_2_ direct hydrogenation catalysts for methanol production using knowledge-based surrogate modeling. *Energy Conversion Management* (2011), 244, 114477.

[16] R. Kajaste, M. Hurme, P. Oinas; Methanol-managing greenhouse gas emissions in the production chain by optimizing the resource base. *AIMS Energy* (2018), 6, 1074–1102.

[17] P. Battaglia, G. Buffo, D. Ferrero, M. Santarelli, A. Lanzini; Methanol synthesis through CO_2_ capture and hydrogenation: Thermal integration, energy performance and techno-economic assessment. *Journal of CO_2_ Utilization* (2021), 44, 101407.

[18] C. M. Jens, L. Muller, L. Kai, A. Bardow; To integrate or not to integrate-techno-economic and life cycle assessment of CO_2_ capture and conversion to methyl formate using methanol. *ACS sustainable Chemistry & Engineering* (2019), 7, 12270–12280.

[19] D. J. Heldebrant, P. K. Koech, V.-A. Glezakou, R. Rousseau, D. Malhotra, D. C. Cantu; Water-lean solvents for post-combustion CO_2_ capture: fundamentals, uncertainties, opportunities, and outlook. *Chemical Reviews* (2017), 117, 9594–9624.

[20] M. Pérez-Fortes, J. C. Schöneberger, A. Boulamanti, E. Tzimas; Methanol synthesis using captured CO_2_ as raw material: Techno-economic and environmental assessment. *Applied Energy* (2016), 161, 718–732.

[21] U. Lee, J. Burre, A, Caspari, J. Kleinekorte, A. M. Schweidtmann, A. Mitsos; Techno-economic optimization of a green-field post-combustion CO_2_ capture process using superstructure and rate-based models. *Industrial & Engineering Chemistry Research* (2016), 55, 12014–12026.

[22] F. Mani, M. Peruzzini, P. Stoppioni; CO_2_ absorption by aqueous NH_3_ solutions: speciation of ammonium carbamate, bicarbonate and carbonate by a ^13^C NMR study. *Green Chemistry* (2006), 8, 995–1000.

[23] B. Xue, Y. Yu, J. Chen, X. Luo, M. Wang; A comparative study of MEA and DEA for post-combustion CO_2_ capture with different process configurations. *International Journal of Coal Science & Technology* (2017), 4, 15–24.

[24] B. Lv, B. Guo, Z. Zhou, J. Jing; Mechanisms of CO_2_ capture into monoethanolamine solution with different CO_2_ loading during the absorption/desorption processes. *Environmental Science & Technology* (2015), 49, 10728–10735.

[25] P. Akula, J. Eslick, D. Bhattacharyya, D. C. Miller; Model development, validation, and optimization of an MEA-based post-combustion CO_2_ capture process under part-load and variable capture operations. *Industrial & Engineering Chemistry Research* (2021), 60, 5176–5193.

[26] D. Higgins, C. Hahn, C. Xiang, T. F. Jaramillo, A. Z. Weber; Gas-diffusion electrodes for carbon dioxide reduction: a new paradigm. ACS Energy Letters (2018), 4, 317–324.

[27] C. M. Gabardo, C. P. O’Brien, J. P. Edwards, C. McCallum, Y. Xu, C.-T. Dinh, J. Li, E. H. Sargent, D. Sinton; Continuous carbon dioxide electroreduction to concentrated multi-carbon products using a membrane electrode assembly. *Joule* (2019), 3, 2777–2791.

[28] A. K. Buckley, T. Cheng, M. H. Oh, G. M. Su, J. Garrison, S. W. Utan, C. Zhu, F. D. Toste, W. A. Goddard, F. M. Toma; Approaching 100% selectivity at low potential on Ag for electrochemical CO_2_ reduction to CO using a surface additive. *ACS Catalysis* (2021), 11, 9034–9042.
